# RNA-sequencing reveals long-term effects of silver nanoparticles on human lung cells

**DOI:** 10.1038/s41598-018-25085-5

**Published:** 2018-04-27

**Authors:** Anda R. Gliga, Sebastiano Di Bucchianico, Jessica Lindvall, Bengt Fadeel, Hanna L. Karlsson

**Affiliations:** 1 Unit of Biochemical Toxicology, Institute of Environmental Medicine, Karolinska Institutet, Stockholm, Sweden; 2 Department of Biochemistry and Biophysics, National Bioinformatics Infrastructure Sweden, Science for Life Laboratory, Stockholm University, Stockholm, Sweden; 3 Division of Molecular Toxicology, Institute of Environmental Medicine, Karolinska Institutet, Stockholm, Sweden

## Abstract

Despite a considerable focus on the adverse effects of silver nanoparticles (AgNPs) in recent years, studies on the potential long-term effects of AgNPs are scarce. The aim of this study was to explore the effects of AgNPs following repeated low-dose, long-term exposure of human bronchial epithelial cells. To this end, the human BEAS-2B cell line was exposed to 1 µg/mL AgNPs (10 nm) for 6 weeks followed by RNA-sequencing (RNA-Seq) as well as genome-wide DNA methylation analysis. The transcriptomics analysis showed that a substantial number of genes (1717) were differentially expressed following AgNP exposure whereas only marginal effects on DNA methylation were observed. Downstream analysis of the transcriptomics data identified several affected pathways including the ‘fibrosis’ and ‘epithelial-mesenchymal transition’ (EMT) pathway. Subsequently, functional validation studies were performed using AgNPs of two different sizes (10 nm and 75 nm). Both NPs increased collagen deposition, indicative of fibrosis, and induced EMT, as evidenced by an increased invasion index, anchorage independent cell growth, as well as cadherin switching. In conclusion, using a combination of RNA-Seq and functional assays, our study revealed that repeated low-dose, long-term exposure of human BEAS-2B cells to AgNPs is pro-fibrotic, induces EMT and cell transformation.

## Introduction

**Figure 1:**
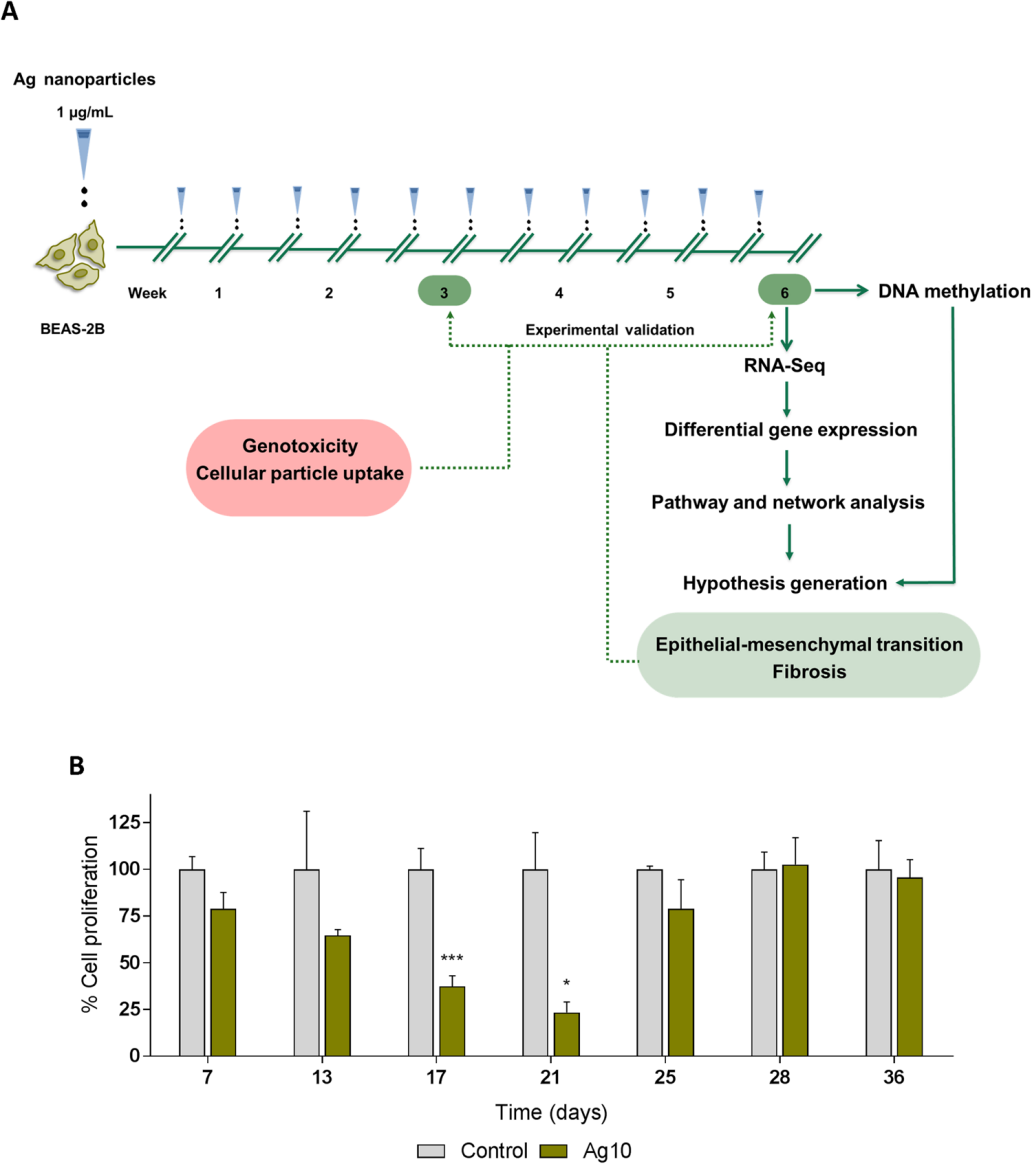
Low-dose, long-term exposure to AgNPs. (**A**) Human BEAS-2B lung cells were exposed to repeated low doses (1 µg/mL) of 10 nm AgNPs for 6 weeks; cells were split and re-exposed twice per week. At the end of the 6-week exposure, RNA-Seq and DNA methylation assays were performed. Bioinformatics analysis of the transcriptomics data concluded with the generation of hypotheses that were experimentally validated at two time-points (3 and 6 weeks) using 10 nm and 75 nm AgNPs. In addition, genotoxicity and NP uptake were assessed. (**B**) Ag10 alters cell proliferation. BEAS-2B cells were exposed to 10 nm AgNPs (1 µg/mL) for 6 weeks. At the indicated time-points, cell proliferation was evaluated by Alamar Blue assay. Results are presented as mean values ± S.D. (n = 3 except day 21, n = 2). *p < 0.05, ***p < 0.001.

The increased production and use of silver nanoparticles (AgNPs) in consumer products and medical devices suggests an increased likelihood of human and environmental exposure to AgNPs. Exposure to AgNPs *via* inhalation is of particular concern, not least in an occupational setting. Consumers can also be exposed to AgNPs, for example when using spray products containing AgNPs^1^. Studies in rodents have revealed that acute inhalation exposure to AgNPs yields minor or short-lived effects on the lungs^2,3^, while for sub-chronic inhalation the main target organs were the lungs and the liver^4^. Size-dependent effects were reported following short-term inhalation of AgNPs, with a moderate pulmonary toxicity induced by the smaller (15 nm) particles, and no observable effects triggered by the larger (410 nm) particles, but all the effects had resolved after one week^5^. In another recent study, the effects of acute, low-dose intratracheal instillation of AgNPs (0.05 μg/g body weight) were examined and the authors noted a reduced lung mechanical function albeit in the absence of any cytotoxicity; these effects resolved after 21 days^6^. Long-term *in vivo* studies are, however, still lacking. In particular, there are no *in vivo* carcinogenicity studies on AgNPs following pulmonary exposure. Similarly, the majority of *in vitro* studies performed on AgNPs have focused on short-term, acute effects, using high doses which have questionable relevance for human exposure. Hence, there is an increasing need for data on the potential long-term effects of AgNPs using experimental designs that more closely mimic real-life exposure scenarios in order to aid risk assessment^7^. In addition, chronic exposure studies are critical for addressing effects such as carcinogenicity, which is a complex, step-wise process unfolding over time. There are only a few instances of long-term *in vitro* studies of nanomaterials, including multi-walled carbon nanotubes^8,9^ titanium dioxide NPs^10^, and AgNPs^11,12^, using the human HaCaT keratinocyte cell line and the human lung bronchial cell line BEAS-2B, respectively. The latter study provided evidence for cell transformation including apoptosis resistance and cell migration/invasion following long-term exposure to AgNPs (100 nm)^12^. In light of the knowledge gaps related to long-term exposure, we designed a repeated, low-dose, *in vitro* study to address the carcinogenic potential of AgNPs. The cell line selected for these studies was BEAS-2B, a non-tumorigenic, SV40 transformed human lung cell suitable for long-term culture and considered a good *in vitro* model for lung carcinogenesis^8,13^. We used AgNPs that were previously studied with respect to short-term exposure^14^. In order to capture the overall impact of long-term, low-dose exposure to AgNPs (Fig. 1A), we utilized next-generation sequencing to examine genome-wide transcriptional changes along with genome-wide DNA methylation analysis to determine whether the transcriptional responses were accompanied by any epigenetic changes. Functional validation of the transcriptomics data was performed using an array of cell-based assays for fibrosis, cell invasion, and other indicators of cell transformation and epithelial-mesenchymal transition (EMT).

## Results

### Characterization of AgNPs

Thorough particle characterization in cell medium with respect to particle size distribution was previously performed and the results showed that AgNPs agglomerate in cell medium, have a multimodal distribution, and sediment with time^14^. The primary particle size and morphology was confirmed by TEM (Supplementary Figure 1A). In addition, zeta potential values of Ag10 and Ag75 were determined in cell medium (Supplementary Figure 1B). We also evaluated the soluble Ag fraction in cell medium following cell exposure. The results showed an average of soluble Ag of 0.2 µg/mL (20 wt%) for Ag10 and 0.14 (14 wt%) for Ag75, although the difference between the two NPs was not significant (Supplementary Figure 1C). In the subsequent study, Ag10 was used for RNA-Seq studies while both Ag10 and Ag75 were examined for cellular uptake and in functional assays.

### AgNPs are taken up by BEAS-2B cells

**Figure 2:**
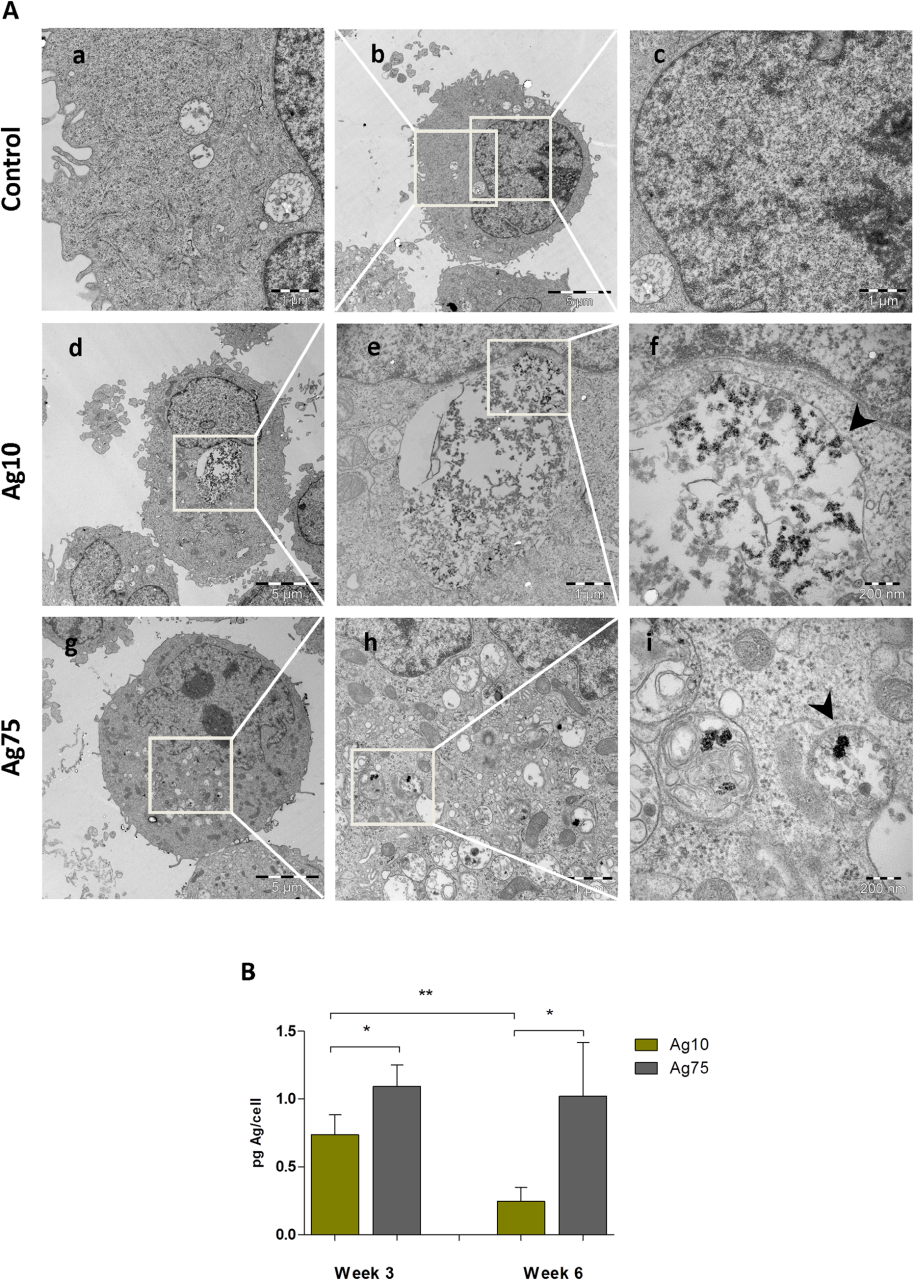
AgNPs are taken up by BEAS-2B cells. (**A**) Intracellular localization of AgNPs after 3 weeks of exposure was investigated by TEM. Both Ag10 and Ag75 nanoparticles were taken up by cells and were localized in membrane bound structures indicated by the arrow-heads (panels f, i). (**B**) Cellular uptake after 3 and 6 weeks of exposure was quantified by ICP-MS. Results and expressed as pg Ag/cell and are presented as mean values ± S.D. (n = 3). *p < 0.05, **p < 0.01.

We determined cellular uptake of AgNPs by BEAS-2B lung cells after 3 weeks of exposure by transmission electron microscopy (TEM) (Fig. 2A). Ag10 localized in the cytoplasm, and were confined to membrane-bound structures suggestive of endo-lysosomal compartments (Fig. 2A, cf. panels f, i). AgNPs were not detected in the cell nucleus. For comparison, we noted a similar subcellular localization pattern for Ag75 (Fig. 2A). In addition, cellular uptake of both NPs was quantified by inductively coupled plasma mass spectrometry (ICP-MS) after 3 and 6 weeks of exposure (Fig. 2B). At both time-points there was a higher Ag content in the Ag75 treated cells as compared to Ag10. The Ag content following Ag10 exposure was reduced with time, from approx. 0.7 pg/cell at week 3 to approx. 0.25 pg/cell at week 6, possibly due to reduced uptake, or increased exocytosis of NPs, whereas the Ag content for cells incubated with Ag75 was constant over time (approx. 1 pg/cell).

### AgNPs affect cell proliferation

Human BEAS-2B cells were exposed to AgNPs (1 µg/mL, or approx. 0.2 µg/cm^2^) for 6 weeks; cells were re-seeded and re-exposed to the AgNPs twice per week. During the 6-week exposure period, cell proliferation was assessed on a weekly basis (Fig. 1B). Ag10 significantly reduced cell proliferation after 17 and 21 days of exposure (37% and 23%, respectively, when compared to control cells) with subsequent recovery, suggesting that phenotypic changes took place in cells exposed to repeated low doses of AgNPs.

### RNA-sequencing reveals changes in gene expression

**Figure 3:**
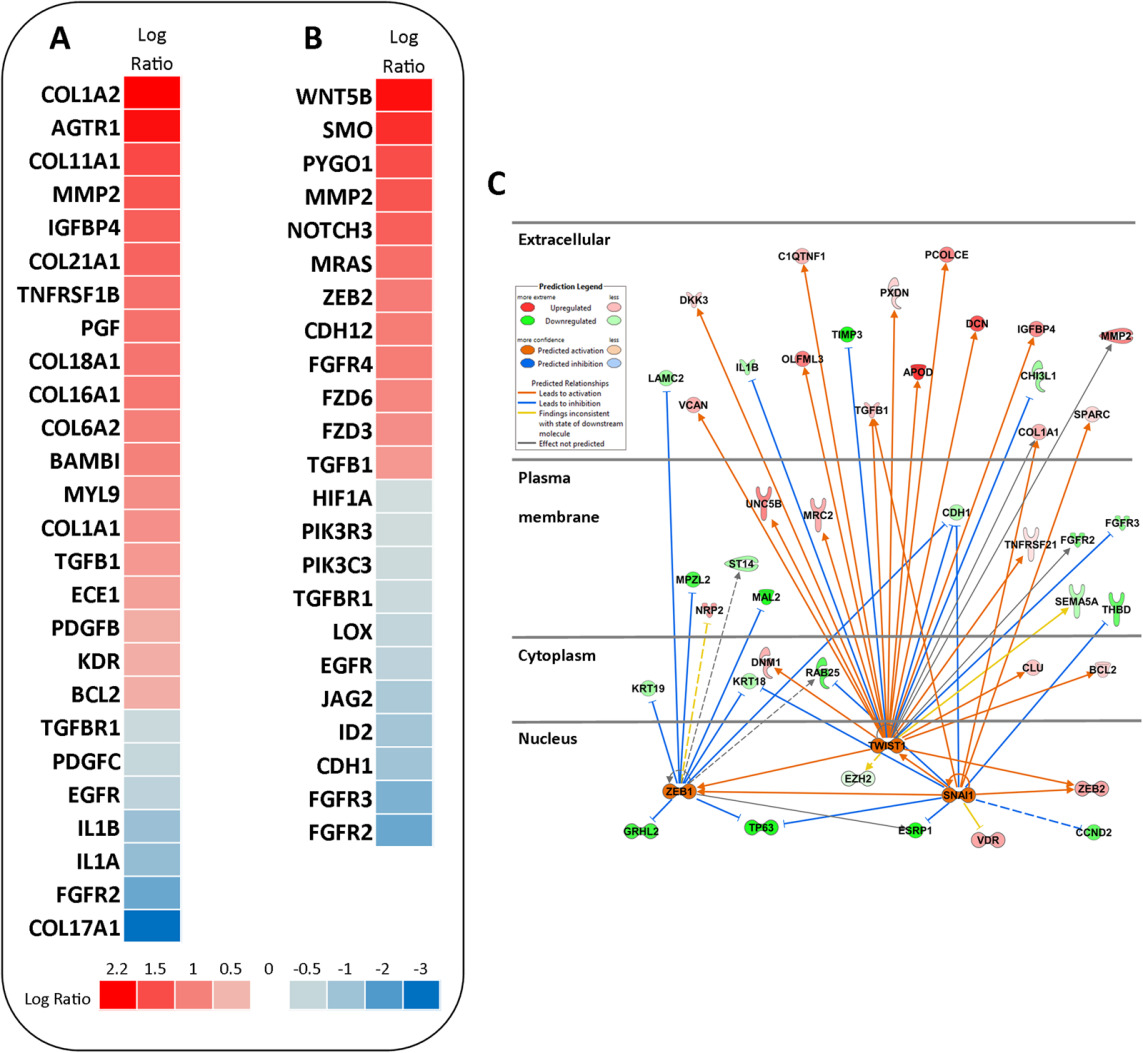
RNA-Seq analysis following long-term exposure to AgNPs. BEAS-2B cells were exposed for 6 weeks to AgNPs at 1 µg/mL and RNA-seq was performed on triplicate samples. Using the IPA tool, pathway analysis of the DEGs was conducted. Heatmaps displaying the gene expression changes in the Ag10 *vs* Control samples were generated for the *hepatic fibrosis* pathway (**A**) and *regulation epithelial-mesenchymal transition* pathway (**B**). (**C**) Upstream regulator analysis was performed using the IPA software tool for Ag10 and a network was generated for the predicted activated transcription factors, *ZEB1*, *TWIST1*, *SNAI1*.

In order to explore the impact of long-term exposure to AgNPs, we performed RNA-Seq using the Hi-Seq. 2500 Illumina platform. The samples (control and Ag10) were sequenced in triplicate and the generated reads were mapped to the human genome version GRCh37. A cut-off for fold change values was not included as we reasoned that the exposure to low doses of nanoparticles might warrant important, but low fold changes in the gene expression. A total of 1717 genes were differentially expressed for Ag10 out of which 719 genes were down-regulated and 998 genes were up-regulated. Next, we performed canonical pathway analysis on the DEGs using the Ingenuity Pathway Analysis (IPA) software. The significantly enriched IPA pathways [−log(p-value > 1.3)] are summarized in Supplementary Table 1. The main aim of the study was to unravel potential carcinogenic effects of AgNPs following low-dose, long-term exposure to AgNPs. Therefore, when exploring the enriched pathways, we focused on pathways related to carcinogenesis including *Hepatic fibrosis pathway* and *Regulation of epithelial-mesenchymal transition pathway*. Heatmaps of the aforementioned pathways are shown in Fig. 3A,B. The *Hepatic fibrosis pathway* is defined by genes related to fibrotic processes in general and is not necessarily liver specific, as the pathway name suggests. Since the present study was performed using lung cells, we examined the altered genes from a lung (pathology) perspective. First, out of the 8 collagen related genes, 7 were up-regulated (*COL1A1, COL1A2, COL6A2, COL11A1, COL16A1, COL18A1, COL21A1*) and only 1 was down-regulated (*COL17A1*). On the other hand, *MMP2*, a matrix-metallopeptidase involved in degradation of collagens (IV, V, VII, X)^15^ was also up-regulated, together with additional matrix-metallopeptidases such as *MMP11*, *MMP19*; the last two were also included in the *Inhibition of metalloproteases pathway* (Supplementary Figure 1D). The expression of *TGFβ1*, an important pro-fibrotic growth factor and a key regulator of lung fibrosis^16^ was up-regulated by Ag10 with the concurrent down-regulation of one of its receptors *TGFBR1* and up-regulation of its pseudoreceptor, *BAMBI*. Several other up-regulated genes in our dataset are correlated with fibrosis and tissue remodeling including angiotensin II receptor type 1 (*AGTR1*)^17^ placental growth factor (*PGF*)^18^, and platelet-derived growth factor subunit B (*PDGF*)^19^.

*Regulation of epithelial-mesenchymal transition* was the second pathway selected for further scrutiny. The gene expression profile suggested cadherin isoform switching, *i.e*., down-regulation of E-cadherin (*CDH1*) and up-regulation of N-cadherin (*CDH12*), which together with up-regulation of *TGFβ1*, *NOTCH3*, and *MMP2* are correlated with EMT^20^. Moreover, *ZEB2*, a transcription factor that mediates EMT by regulating *CDH1*^21^, was also up-regulated by Ag10. In addition, expression of *MRAS*, a gene which was up-regulated in our dataset, is correlated with EMT and oncogenic transformation^22^. On the other hand we found that the gene encoding hypoxia inducible factor-1α (*HIF1α*), which is known to promote EMT *via* ZEB1, among others^23^, was down-regulated. Other pathways closely connected with the EMT pathway are the *Wnt/βcatenin pathway* and the *Wnt/Ca*^*2*+^ *pathway*^20,24^, both altered in our dataset (Supplementary Table 1, Supplementary Figure 2A,B).

Oxidative stress is a common mechanism by which NPs may exert toxicity^25^. NRF-2 mediated oxidative stress response was included among the pathways to reach significance in our dataset (Supplementary Table 1) with a positive z-score (0.8) which is indicative of pathway activation. The pathway was defined by up-regulation of gene expression of antioxidant enzymes such as glutathione-S-tranferases (*GSTM1, GSTM2*, *GSTM3*, *GSTT2/GSTT2B*), enzymes involved in clearing lipid peroxidation products, oxidized DNA bases and endogenous oxidation compounds^26^, NAD(P)H:quinone acceptor oxidoreductase 1 (*NQO1*), an inducible multifunctional antioxidant enzyme, and epoxy hydrolase *EPHX1* (Supplementary Figure 1E). On the other hand, the gene expression of catalase (*CAT*), an antioxidant enzyme, and several mitogen-activated kinases (*MAP3K1*, *MAPK5*, *MAPK14*), which are known to be activated by ROS^27^, were down-regulated in cells exposed for 6 weeks to Ag10 (Supplementary Figure 1E).

To further explore the DEGs, we performed gene ontology (GO) enrichment analysis. The top-20 enriched ontologies for each category are displayed in Supplementary Table 3. Among the most significant ‘biological process’ ontologies were *cell adhesion*, *extracellular matrix organization*, *collagen metabolic process, cell migration* in line with processes identified by the IPA pathway analysis. At the ‘cellular component’ level, *the cytosol*, *extracellular matrix*, *lysosome*, *cytoplasmic membrane-bounded vesicle* and *cell junction* were among the enriched ontologies. At the ‘molecular function’ level the top ontologies were *protein binding*, *glycosaminoglycan binding*, *metal ion binding* and *sulfur compound binding*. The latter results could provide indications regarding the mechanism of action of AgNPs following long-term exposure. It is well established that Ag binds to proteins due to a high affinity for thiol groups, and it was recently demonstrated, on the basis of synchrotron radiation analytical techniques, that dissolution and speciation of Ag mediates the cytotoxicity of AgNPs in THP-1 monocytic cells^28^.

Finally, we analyzed the transcriptomics data using the IPA software to identify upstream transcriptional regulators that could potentially help to explain the observed gene expression changes^29^. The results of the upstream regulator analysis for Ag10 are reported in Supplementary Table 2, and a network around a selection of predicted activated transcription factors (z-score > 2) known to be involved in EMT (*ZEB1*, *SNAI1*, *TWIST1*)^20^ is depicted in Fig. 3C. Taken together, the RNA-Seq analysis revealed several interesting findings including the perturbation of pathways related to epithelial-mesenchymal transition or EMT^20^.

### Marginal effects on DNA methylation

To ascertain whether the gene expression changes induced by Ag10 were linked to differences in DNA methylation, we performed genome-wide DNA methylation array analysis using the Infinium HumanMethylation450 BeadChip. We found only one differentially methylated gene promoter, corresponding to a poorly characterized gene (ENSG00000250358), 6 differentially methylated CpG sites and 5 differentially methylated tiling regions (Supplementary Table 4). Most sites/tiling regions were located in intergenic regions with regulatory function (containing histone acetylation marks or binding sites for transcription factors). Interestingly, we identified two transcription factors (SP1 and STAT3) that were predicted as upstream regulators of gene expression (absolute z-score > 1.5) which also bind differentially methylated sites/regions identified in our DNA methylation array (Supplementary Figure 3). Ag10 thus seemed to alter the DNA methylation pattern in regulatory regions, although it is unclear to which extent these minor changes contributed to the observed changes in gene expression.

### Functional validation of RNA-Seq results

**Figure 4:**
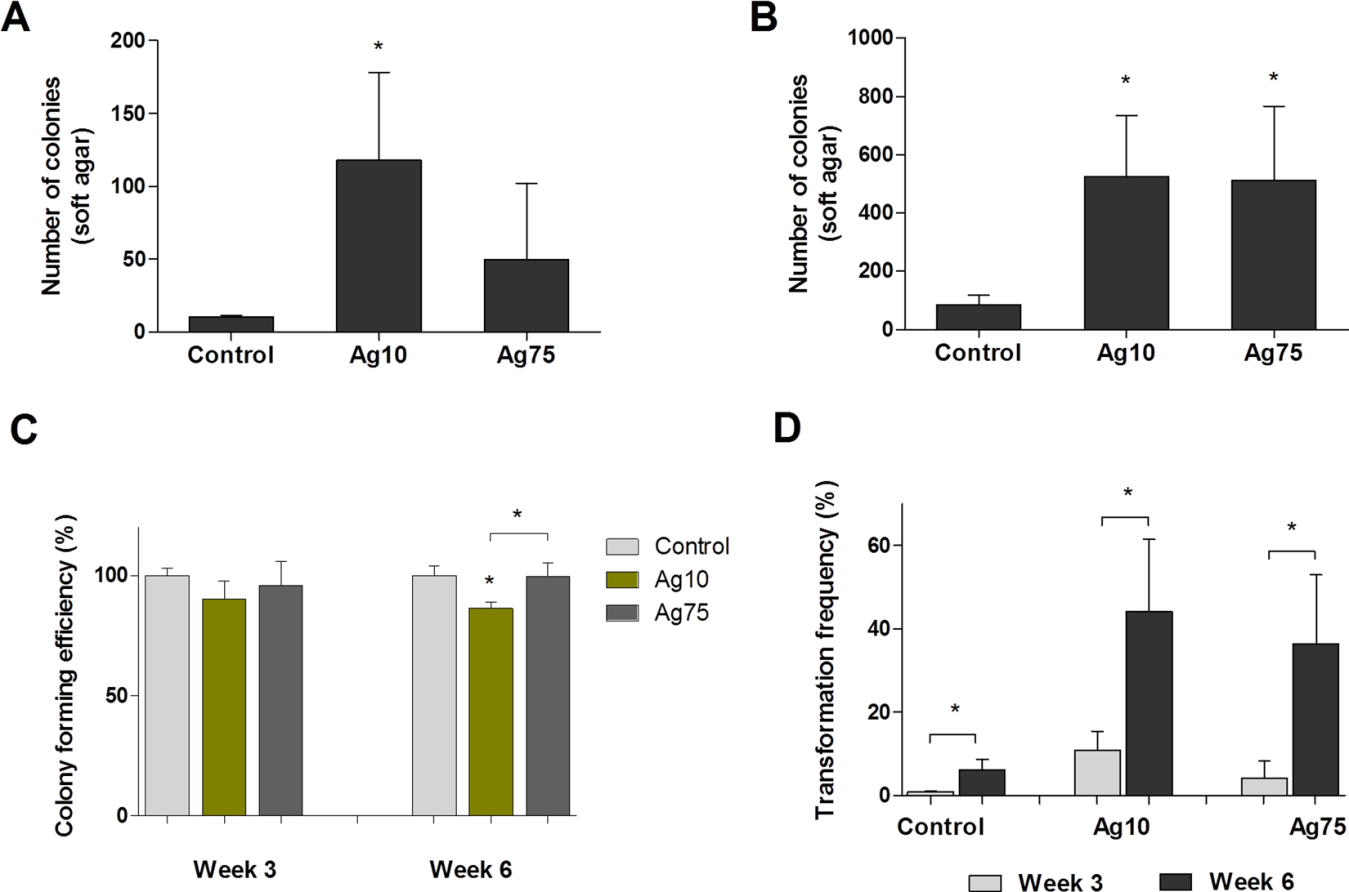
AgNPs induce cell transformation in BEAS-2B cells. Soft agar cell transformation and colony forming efficiency was determined after 3 and 6 weeks of exposure to 10 nm and 75 nm AgNPs (1 µg/mL). Results are expressed as total number of colonies in soft agar after 3 weeks (**A**) and 6 weeks (**B**). The colony forming efficiency results (**C**) were used to calculate the overall transformation frequency (**D**). Results are shown as mean values ± S.D., n = 3 (*p < 0.05).

**Figure 5:**
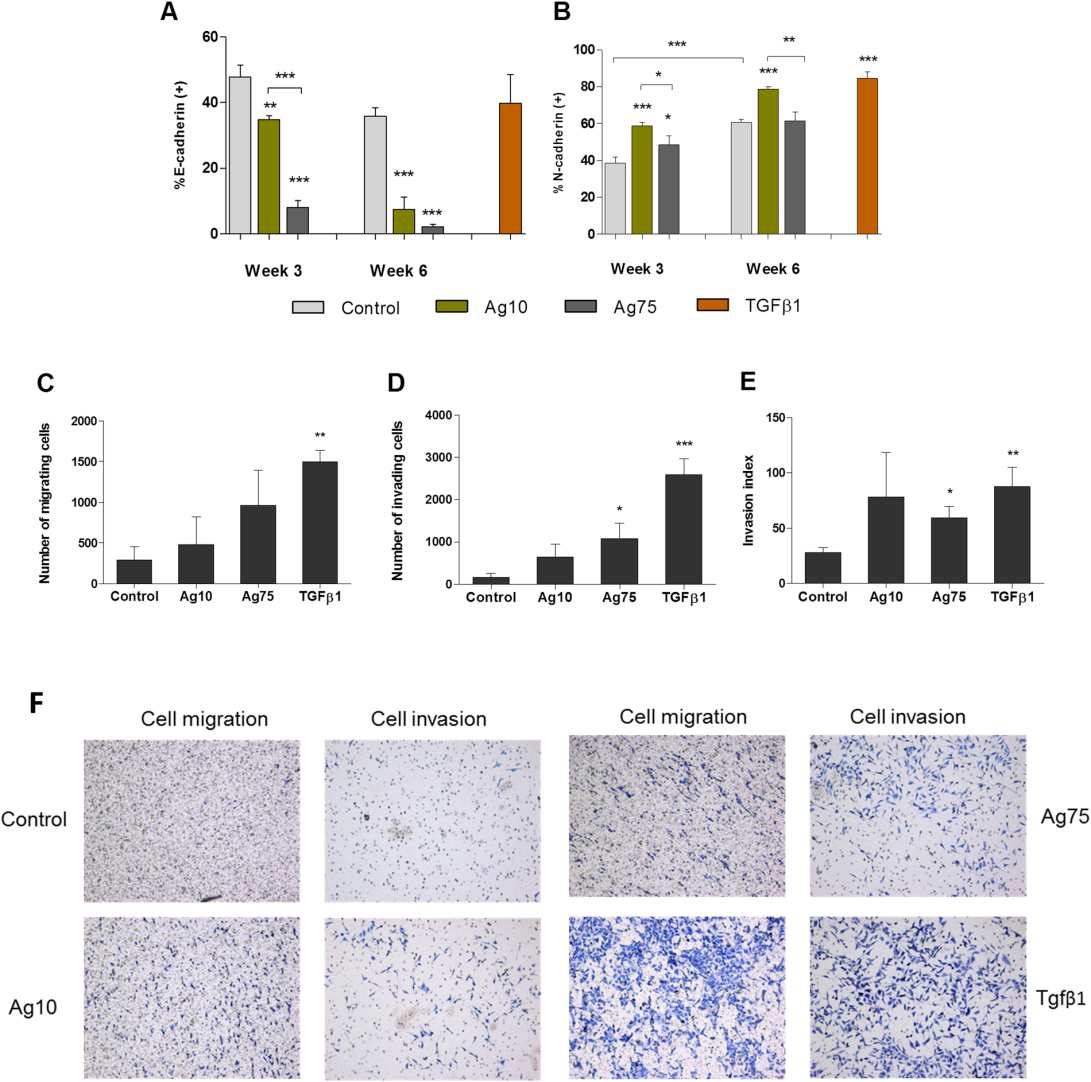
AgNPs induce epithelial-mesenchymal transition in BEAS-2B cells. (**A**,**B**) E-cadherin and N-cadherin surface expression after 3 and 6 weeks of exposure to AgNPs was estimated by flow cytometry. Results are presented as % E-cadherin (**A**) or N-cadherin (**B**) positive cells (mean values ± S.D., n = 3). (**C**–**E**) Cell migration and invasion was evaluated after 6 weeks of exposure to AgNPs (1 µg/mL). Results are expressed as the number of migrating (**C**) and invading (**D**) cells as well as invasion index (**E**) (mean values ± S.D., n = 3). Positive control: TGF-β1 15 ng/mL 72 h. Significant results are marked with asterisks (*p < 0.05, **p < 0.01, ***p < 0.001). (**F**) Representative images from the cell migration and cell invasion assays.

On the basis of the RNA-Seq data analysis, we selected two pathways (*Hepatic fibrosis*, and *Regulation of the epithelial-mesenchymal transition*) and designed validation experiments. Furthermore, we also performed genotoxicity assays at 3 weeks and 6 weeks of exposure. In order to elucidate whether the effects observed in the functional assays were specific for Ag10, or more generally relevant for AgNPs, we also included 75 nm-sized AgNPs in these studies. Our pathway analysis of the RNA-Seq results revealed that Ag10 altered the *Regulation of the epithelial-mesenchymal transition pathway* in a manner consistent with induction of EMT. EMT is a complex process associated with cancer progression in which epithelial cells acquire a mesenchymal phenotype characterized by increased cell motility, invasive behavior, and cadherin switching^30^. In order to validate the RNA-Seq findings, we evaluated the cell transformation, cellular migration, and invasion potential, together with the expression of epithelial (E-cadherin) and mesenchymal (N-cadherin) markers after long-term exposure of BEAS-2B to AgNPs. First, we investigated the cell transformation potential of AgNPs by assessing the anchorage-independent cell growth in soft agar, which is a hallmark of carcinogenesis^31^. After 3 weeks, Ag10, but not Ag75, increased the number of soft agar colonies (Fig. 4A), whereas after 6 weeks both NPs increased colony growth (Fig. 4B). The colony forming efficiency (CFE) was somewhat reduced for Ag10 at week 6 (Fig. 4C). The transformation index was calculated based on plating efficiency and clonogenicity as evidenced by the CFE assay and was found to increase over time for both AgNPs (8.1 fold for Ag10, 17.5 fold for Ag75) (Fig. 4D). Representative images from the CFE assay are shown in Supplementary Figure 5. Next, we examined the surface expression of E-cadherin (Fig. 5A) and N-cadherin (Fig. 5B) using TGFβ1 as a positive control. At week 3 and week 6, Ag10 and Ag75 reduced the % of cells expressing E-cadherin. After 3 weeks of exposure, Ag75 had a stronger effect compared to Ag10, while the effects were more similar between the two NPs at 6 weeks of exposure. Ag10 increased the % of cells expressing N-cadherin both at week 3 and week 6, while Ag75 had an effect only after 3 weeks. For this surface marker, TGFβ1 was proven to be a robust positive control. In addition, N-cadherin expression in the untreated cells increased with time from approx. 40% at week 3 to approx. 60% at 6 weeks. We also investigated the migration and invasion potential following exposure for 6 weeks and found that there was an increasing trend (not significant) of cell migration (Fig. 5C), and a significant increase in cell invasion for Ag75 (Fig. 5D). The invasion index (% of migrating cells that are able to invade), increased for both AgNPs, but this was significant only for Ag75 (Fig. 5E). TGFβ1 was included as a positive control. Figure 5F shows representative cell migration/invasion results.

**Figure 6:**
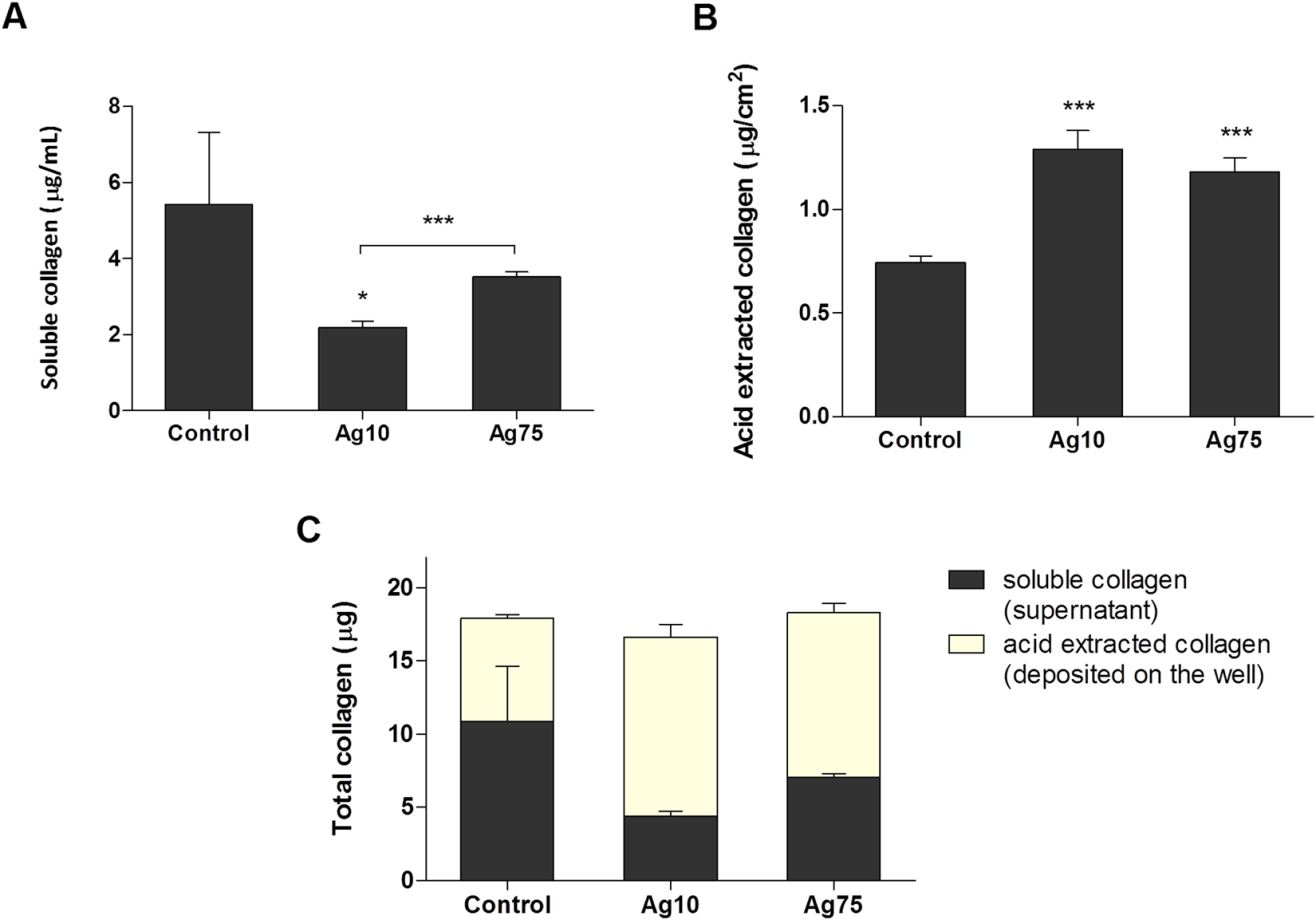
AgNPs increase collagen deposition in BEAS-2B cells. Collagen production after 6 weeks of exposure (5 days after final re-seeding) was quantified using the Sircol assay. Soluble collagen secreted in the cell medium (**A**) was expressed as µg/mL, acid extracted collagen (insoluble collagen deposited on the well plate) (**B**) was expressed as µg/mL and total collagen (C) was expressed as µg. Results are presented as mean values ± S.D. (n = 3). *p < 0.05, ***p < 0.001.

Lung fibrosis is characterized by extracellular matrix remodeling and is a well-established toxic response to particle exposure which could play a role in particle-induced carcinogenesis^32^. Our pathway analysis of the RNA-Seq data indicated an alteration of the *Hepatic fibrosis pathway* which was defined by the up-regulation of a large number of collagen related genes. We therefore proceeded to determine total collagen secretion and deposition on cell culture plates after 6 weeks of AgNPs exposure (5 days from the last re-seeding). The amount of soluble collagen in cell medium after 5 days of culture was reduced after exposure to Ag10 and Ag75, with Ag10 having a more marked effect (Fig. 6A). On the other hand, both AgNPs increased the acid soluble collagen, *i.e*., newly formed and deposited collagen, to a similar extent (approx. 1.3 µg/cm^2^ acid extracted collagen for Ag10 and approx. 1.2 µg/cm^2^ for Ag75 *vs* approx. 0.7 µg/cm^2^ for the control) (Fig. 6B). There was no difference in the total amount of collagen between the conditions (Fig. 6C). In sum, the data suggests that AgNPs interfere with collagen secretion kinetics and collagen deposition, indicative of a pro-fibrotic potential of these NPs.

**Figure 7:**
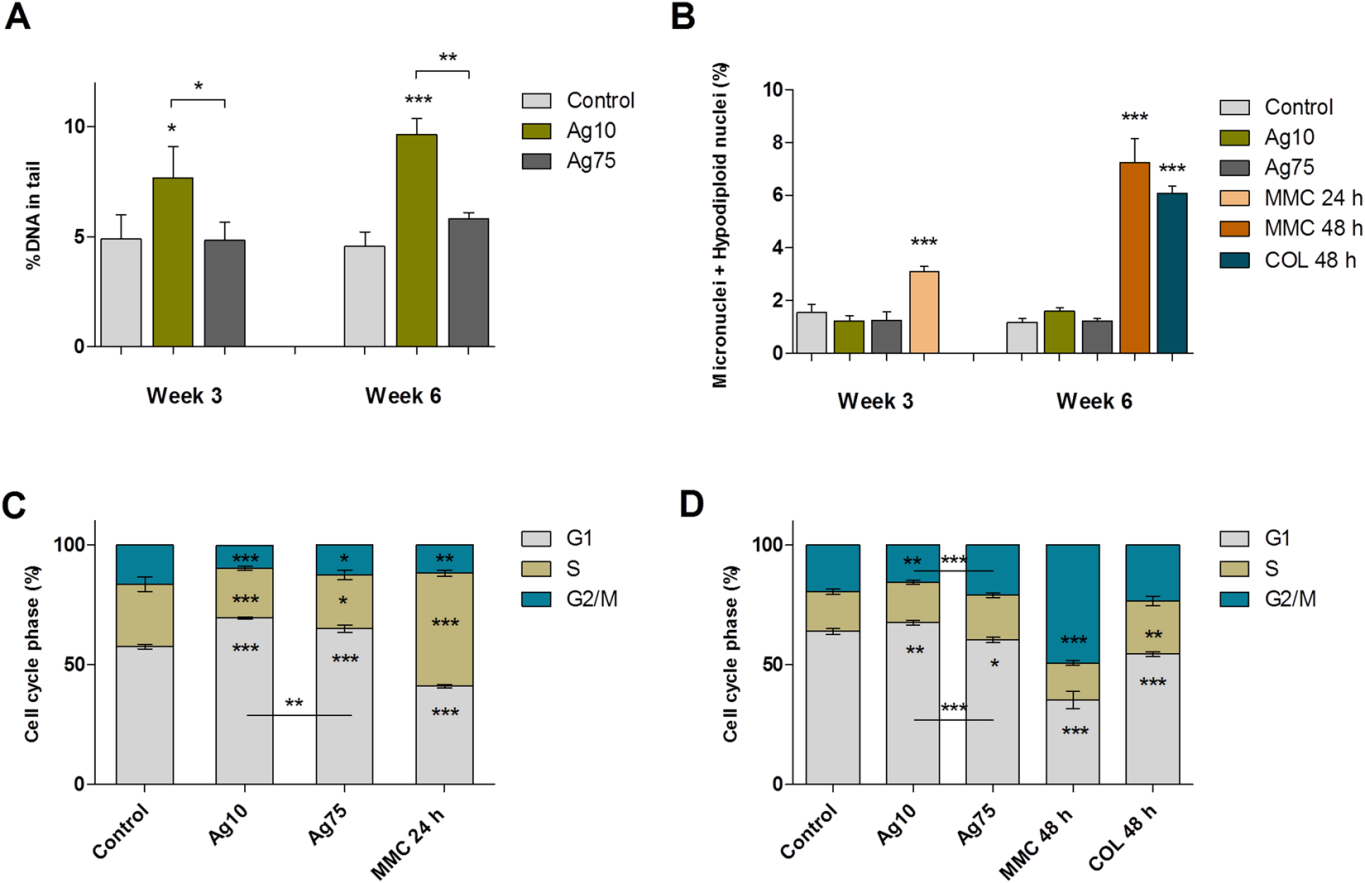
AgNPs cause DNA strand breaks and cell cycle arrest in BEAS-2B cells. (**A**) DNA damage induction after 3 and 6 weeks of exposure to AgNPs (1 µg/mL) was investigated using the alkaline comet assay. Results are expressed as % DNA in tail and presented as mean values ± S.D. (n = 3). (**B**) Micronuclei formation after 3 and 6 weeks of exposure to AgNPs (1 µg/mL) was evaluated by flow cytometry. Results are expressed as percentage of micronuclei and hypodiplodid nuclei (%) and presented as mean values ± S.D. (n = 3). Cell cycle phase evaluation was performed by flow cytometry after 3 weeks (**C**) and 6 weeks (**D**) of exposure to AgNPs (1 µg/mL). Results are presented as mean distribution (%) of the different cell cycle phases (G1, S, G2/M) ± S.D. (n = 3). *p < 0.05, **p < 0.01, ***p < 0.001. Mytomycin C (MMC), 0.05 µg/mL 24 h or 48 h; and demecolcine (COL) 0.01 µg/mL 48 h served as positive controls.

Finally, due to the relevance of genotoxicity for the carcinogenic potential of particles, we performed alkaline comet assay (Fig. 7A) and the micronucleus test (Fig. 7B) following 3 and 6 weeks of low-dose exposure of BEAS-2B to AgNPs. A flow cytometry version of the micronucleus test was used^33^, in order to concurrently assess cytotoxicity and cell cycle alterations. The alkaline comet assay revealed that Ag10 induced a significant increase in DNA damage both after 3 and 6 weeks of exposure. No effect was observed for Ag75. Moreover, neither of the AgNPs induced micronuclei or hypodiploid nuclei formation. Mitomycin C and demecolcin were included as positive controls. We also analyzed the distribution of the different phases of the cell cycle after exposure of BEAS-2B cells for 3 (Fig. 7C) or 6 weeks (Fig. 7D). The data indicated G1 arrest mainly for lung cells exposed to Ag10 when compared to untreated cells; the cell cycle arrest was more pronounced at 3 weeks.

## Discussion

Several *in vitro* studies have provided evidence for size- and coating-dependent cyto- and genotoxicity of AgNPs in various cell models^14,34–37^. Cells were typically exposed up to several days. Furthermore, previous studies have indicated that AgNPs could trigger DNA damage *in vivo* following inhalation exposure or intravenous injection in rats or mice^38–40^, but there are, as yet, no chronic studies on the potential *in vivo* carcinogenicity of AgNPs. To our knowledge, only a small number of *in vitro* studies have addressed low-dose, long-term effects of AgNPs in relation to the carcinogenic potential of these materials^12,41^. In the present study, we applied ‘omics’ approaches, *i.e*., RNA-Seq and DNA methylation array-based analysis, coupled with conventional toxicological assays, in order to gain a comprehensive understanding of the potential low-dose effects of AgNPs. Overall, our data suggested that AgNPs have pro-fibrotic potential and trigger EMT as well as cell transformation in human lung cells exposed to low doses for 6 weeks.

In the present study, we performed RNA-seq of BEAS-2B cells exposed for 6 weeks to 10 nm-sized AgNPs. In the following validation experiments, new long-term experiments were performed using 10 nm and 75 nm AgNPs. The same NPs were previously studied with respect to short-term (up to 24 h) exposure in BEAS-2B cells^14^, and the 10 nm particles were found to be more cytotoxic than the 75 nm particles (up to 50 µg/mL); however, both particles triggered DNA damage as evidenced by the comet assay. Here, the same BEAS-2B cell line was exposed to a low dose (1 µg/mL or approx. 0.2 µg/cm^2^) for 3 or 6 weeks. One may ask: what is a *low dose*? It is difficult to translate the dose used in the present *in vitro* study to human exposure; however, we offer some considerations here regarding the study design. Human health studies with AgNPs are scarce and the reported exposure from personal sampling in manufacturing factories ranges from 0.12–1.02 µg/m^3^ ^42^. However, maximum levels of up to 289 µg/m^3^ were detected in the injection room of a manufacturing plant^43^. If we consider this latter peak exposure and the assumptions reported by Wang *et al*.^44^ (*i.e*., ventilation rate 20 L/min, deposition fraction 30%, monthly exposure of 8 h/day, 5 days a week for 4 weeks, human alveolar surface area 102 m^2^/person) then 0.2 µg/cm^2^ is equivalent to the lung deposition after approx. 12 months of exposure. We have not considered the total administered dose in our cell model because our ICP-MS data indicated that there was no Ag accumulation in BEAS-2B cells over time. In addition, the above estimation is focused on the total deposited mass per cm^2^ and does not take into account the deposition pattern of the particles. Nevertheless, it has been reported that particles could concentrate a few hundred times at the airway carinas, forming so called ‘hot spots’, which is believed to be the originating point for bronchogenic carcinomas^45^. Overall, therefore, the doses used here may be considered relevant for human exposure to AgNPs in an occupational setting. Other recent *in vitro* studies of ‘low-dose’ effects of nanomaterials have used doses in the range of 0.02–4.0 µg/cm^2^ (MWCNTs)^8,9^, 1–20 µg/mL (TiO_2_ NPs)^10^, 0.05–0.1 µg/mL (Co NPs)^46^, and 0.13–1.33 µg/mL for AgNPs in BEAS-2B cells^12^. Comfort *et al*.^11^ exposed HaCaT keratinocytes to exceedingly low concentrations of AgNPs (pg/mL), but the authors incorporated an adjustment factor to account for the fact that only a few % of all AgNPs, regardless of the route of exposure, are retained in the skin. Finally, it should be noted that *long-term* exposure in the present study refers to repeated exposure over 6 weeks, since the cell cultures were re-seeded twice weekly; hence, the exposure was repeated as opposed to chronic.

BEAS-2B cells are considered a good model for long-term studies of carcinogenesis induced by heavy metals and NPs (studied performed up to 6 months)^8,13^. However, during long-term exposure a change in cell phenotype and even cell transformation may occur as a result of cell culture *per se*. During our experimental validation we observed that parameters such as anchorage independent cell growth and N-cadherin expression increased with time in the unexposed cells, indicating potential spontaneous cell transformation. In addition, there was a change in the distribution of the cell cycle phases in the control. On the other hand, the background level of DNA damage as measured by the comet assay and micronucleus test did not change with time in culture, suggesting both genomic and chromosomal stability. Nevertheless, the effects elicited by a 6-week exposure to AgNPs were pronounced.

Size-dependent toxicity of AgNPs has previously been reported both *in vitro* and *in vivo*^5,14,44^. In the present study, we also identified a series of endpoints for which there was a clear size-dependent effect. For example Ag10 induced DNA damage as evidenced in the comet assay results and had more pronounced effect on the cell cycle (arrest in G1). However, there was no indication of nanoparticles in the nucleus and therefore a direct effect on DNA seems unlikely. For other endpoints such as cell invasion and reduction of E-cadherin (but not increase in N-cadherin) Ag75 seemed to be more potent. Thus, it appears possible that size is important for different endpoints in various ways, which has been noted previously for Ag nanoparticles^47,48^.

The use of ‘omics’ technologies coupled with appropriate computational approaches to determine statistically significant perturbations of genes or pathways represents an attractive method to identify the potential hazards and mechanisms of action of chemicals and nanomaterials^49^. In the present study, we applied RNA-seq, a method that provides a far more detailed measurement of levels of transcripts than other approaches^50^, and we found that 1717 genes were differentially expressed in BEAS-2B cells exposed to 1 µg/mL Ag10 of which 719 genes were down-regulated and 998 genes were up-regulated. Next, we performed pathway analysis using the IPA software. After initial exploration of the results, we selected two pathways for functional validation, the ‘fibrosis’ and ‘epithelial-mesenchymal transition’ (EMT) pathway, based on their relevance for carcinogenesis. Due to the link between genotoxicity and carcinogenesis, genotoxicity assays were also performed. Our experimental validation showed that both Ag10 and Ag75 induced EMT in BEAS-2B cells with cadherin switching and increased cell invasion in a matrigel invasion assay. In addition, the AgNPs also increased anchorage-independent cell growth, a marker of cell transformation^31^. Thus, we could validate the RNA-seq based predictions using functional assays. Confirmation of the up- or down-regulation of individual genes or proteins is useful in terms of anchoring ‘omics’ results. For instance, in a recent microarray study, we observed that the gene encoding monocyte chemoattractant protein 1 (MCP1, also known as CCL2) was 88-fold up-regulated in the lungs of rats exposed to CuO NPs, in line with the histopathological findings, and we could verify this by immunohistochemical detection of CCL2 in the lungs^51^. However, pathway analysis affords a more comprehensive view of the transcriptomics data. Kinaret *et al*.^52^ recently investigated transcriptomic responses of the human THP-1 monocytic cell line *versus* lung tissues of mice after exposure to carbon-based nanomaterials, and they could not observe significant commonalities at the level of individual genes. However, when focusing on the biological functions affected by nanomaterials *in vitro* and *in vivo*, the authors could identify shared patterns of gene regulation^52^. In the present *in vitro* study, we aimed to perform *functional* validation of the transcriptomics results, focusing on pathways and end-points that are known to play a role in carcinogenesis, and we could successfully corroborate our RNA-seq results, not least the fact that long-term, low-dose exposure of human lung cells to AgNPs triggers EMT. Only a few studies have investigated EMT induction following exposure to nanomaterials. Two recent studies showed that multi-walled carbon nanotubes can induce EMT both *in vitro* and *in vivo*^53,54^. Another recent study, published as the present study was underway, also provided evidence that long-term exposure to AgNPs induces cell transformation in BEAS-2B cells^12^. In a very recent study using the colon adenocarcinoma Caco-2 cell line, AgNPs were found to elicit signs of cell transformation after a 6-week, low-dose (0.5–1.0 µg/mL) exposure^41^. However, carcinoma cell lines may not be suitable models for studies of malignant transformation. Our RNA-Seq based approach indicated that AgNPs not only induced EMT, but also markers of fibrosis; the strength of global transcriptomics approaches is that unanticipated effects can also be revealed. Nevertheless, despite using different approaches and different cell culture conditions, our results and those of Choo *et al*.^12^ are largely in agreement.

TGFβ is not only a mediator of EMT^20^, but also a potent pro-fibrogenic factor^54^. Recent *in vitro* studies demonstrated that long-term exposure to sub-toxic doses of multi-walled carbon nanotubes induced EMT *via* a TGFβ-dependent signaling pathway, and *in vivo* studies supported the occurrence of pulmonary fibrosis following MWCNT exposure^54^. Mild pulmonary fibrosis has also been reported following exposure to AgNPs along with an increase in TGF-β in bronchoalveolar lavage fluid^44^. In the present study, pathway analysis of the RNA-seq results indicated that the (hepatic) fibrosis related pathway was perturbed in BEAS-2B cells exposed for 6 weeks to AgNPs. Subsequent experiments showed that although the total amount of collagen was unchanged, AgNPs altered the collagen kinetics in BEAS-2B cells by increasing collagen deposition, a sign of pro-fibrotic potential. Collagen deposition and extracellular matrix stiffness can contribute to cell invasion and other hallmarks of cancer^55^. We did not find an upregulation of TGF-β production in BEAS-2B cells at 6 weeks of exposure (data not shown). Furthermore, the AgNPs-induced collagen deposition occurred in the absence of a classical inflammatory response (as evidenced by the down-regulation of the acute phase response pathway), suggesting an alternative mechanism. Further studies are warranted to explore the involvement of fibrosis in the long-term carcinogenic effects of AgNPs.

Environmental agents can exert epigenetic effects and there is increasing evidence of epigenetic mediation of gene expression in human diseases^56^. Studies investigating epigenetic changes as a consequence of long-term exposure to NPs are scarce. However, a recent study reported that MWCNTs but not TiO_2_ induced changes in DNA methylation after 4 week exposure in BEAS-2B cells, despite no nanomaterial uptake^57^. These changes were mainly associated with hypomethylation and they were localized predominantly on chromosome X^57^. In our study we found marginal effects on DNA methylation, and the changes were associated with regulatory regions. It remains to be explored to which extent these effects contribute to the oncogenic phenotype observed in our study.

## Conclusions

We used next-generation sequencing combined with a suite of conventional toxicological assays to decipher the biological pathways altered by long-term exposure of lung cells to AgNPs. Taken together, our results pointed towards induction of fibrosis, EMT, and cell transformation indicative of an oncogenic phenotype, following long-term exposure to low doses of AgNPs. We also observed induction of DNA strand breaks for Ag10 at 3 and 6 weeks, but not for Ag75. These findings suggest potential human health hazards following chronic exposure to AgNPs and highlight the use of long-term *in vitro* studies for studying effects related to carcinogenicity of nanoparticles. The present study has also demonstrated that RNA-Seq combined with appropriate validation studies can provide valuable insights into the toxicity of nanomaterials.

## Materials and Methods

### Nanoparticles

The nanoparticles used in the present study were 10 nm and 75 nm citrate-coated AgNPs (BioPure). Both materials were purchased from Nanocomposix (San Diego, CA) (stock: 1 mg/mL in aqueous 2 mM citrate). Particle characterization in cell medium (BEGM) in terms of primary size (electron microscopy), hydrodynamic size (photon cross correlation spectroscopy), and silver released in cell medium (atomic absorption spectroscopy) was previously reported^14^. Zeta potential values were determined using a Malvern Zetasizer Nano ZS.

### BEAS-2B cell culture conditions

BEAS-2B cells (European Collection of Cell Cultures) were cultured in bronchial epithelial cell growth medium (BEGM, Lonza) supplemented with BEGM bullet-kit (Lonza: recombinant epidermal growth factor (EGF), hydrocortisone, insulin, bovine pituitary extract, GA-1000 (Gentamicin Sulfate and Amphotericin-B), retinoic acid, transferrin, triiodothyronine and epinephrine). All experiments were conducted in accordance with relevant guidelines. Cells were cultured in flasks and plates pre-coated with: 0.01 mg/mL fibronectin, 0.03 mg/mL bovine collagen type I, 0.01 mg/mL bovine serum albumin and 0.2% penicillin-streptomycin in BEGM additive free medium for 1-2 h prior to the seeding. Cells were maintained in a humidified atmosphere at 37 °C, 5% CO_2_ and sub-cultured at 80% confluency. For long-term exposure, BEAS-2B were seeded in 6 well-plates (5 × 10^3^ cells/cm^2^, 2 mL cell medium per well) and allowed to attach for approx. 2 h. Thereafter, cells were exposed to 1 µg/mL Ag10 or Ag75 (approx. 0.2 µg/cm^2^) in triplicates. Cells were split (trypsinization), counted, re-seeded twice a week (5 × 10^3^ cells/ cm^2^, 2 mL cell medium per well) and re-exposed to the AgNPs. The experiments in this study are derived from two series of experiments: cell proliferation, RNA-Seq and DNA methylation assays were performed in the first series, and in the second series, we performed functional validation experiments, described below.

### Transmission electron microscopy

The cellular uptake and intracellular localization of the nanoparticles were visualized by TEM. After 3 weeks of exposure cells were harvested by trypsinization and fixed in freshly prepared 2.5% glutaraldehyde in 0.1 M phosphate buffer (PB). After fixation the pellet was rinsed in 0.1 M PB and post fixed in 2% osmium tetroxide in 0.1 M PB, pH 7.4 at 4 °C for 2 h, dehydrated in ethanol followed by acetone, and embedded in LX-112 (Ladd). Ultrathin sections (approximately 60–80 nm) were cut by a Leica ultracut UCT (Leica) and contrasted with uranyl acetate followed by lead citrate and examined with Tecnai 12 Spirit Bio TWIN transmission electron microscope (Fei) at 100 kV. Digital images were captured by using a Veleta camera (Olympus Soft Imaging Solutions).

### Inductively coupled plasma mass spectrometry

Cellular uptake of the AgNPs was quantified using ICP-MS. After 3 and 6 weeks of exposure, cells were washed three times with PBS, harvested by trypsinization, re-suspended in cell medium and counted. The mineralization of the samples was performed in 4% HCl and 40% HNO_3_ for 48 h. Thereafter the samples were diluted to reach 2% HNO_3_ prior to the analysis. ^107^Ag and ^109^Ag isotopes were quantified using an iCAP Q instrument (Thermoscientific) running on KED mode. Calibration standards of 1, 5, 10, 50, 100, 500 ppb Ag were prepared using a 1000 ppm reference standard (Spectrascan). All samples were spiked with 5 ppb indium as an internal standard with a range of recovery between 85–105%. The limits of detection for the investigated isotopes were estimated at < 0.004 ppb for both isotopes. Each sample was injected at least 3 times and the relative standard deviation (RSD) acceptance was set at 10%. The average values of the analyzed isotopes were used and results were expressed as pg Ag/cell. For the Ag release in cell medium cell supernatants (4 days after exposure) were collected, spun down (15000 rpm, 30 min) and the upper fraction was mineralized and analyzed as described above.

### Nucleic acid extraction

At the end of the 6-week exposure, total RNA was extracted using the RNeasy Mini Columns (Qiagen) in accordance with manufacturer’s instructions including the purification step with DNase I. Total RNA concentration was determined using NanoDrop (NanoDrop Technologies). Samples were submitted in triplicates for RNA-Seq analysis. The quality control of the RNA samples was conducted using the Bioanalyzer 2100 (Agilent Technologies) and all samples had RIN values above 8. For the DNA methylation assay, DNA was extracted after 6 weeks of exposure using the Invisorb Spin Tissue Mini Kit (Stratec) in accordance with the manufacturer’s instructions. DNA concentration was determined using NanoDrop. DNA methylation analysis was performed on triplicate samples as described below.

### RNA-sequencing and data analysis

RNA sequence libraries were generated with standard mRNA stranded protocols from Illumina and sequenced on a Hiseq. 2500 (pair end reads 101 bp long, RapidRun mode) at the Science for Life Laboratory, Stockholm, Sweden. Data processing was carried out at SNIC-UPPMAX, Uppsala, Sweden^58^. The generated reads were mapped to the human genome version GRCh37 using Tophat v. 2.0.4^59^. Read data were converted to gene counts with the program htseq v. 0.6.1^60^. Differential gene expression was assessed using Bioconductor DESeq. 2 package^61^ running in R language version 3.2.0. Only genes with p-values lower than 0.05 after FDR correction for multiple testing were considered differentially expressed genes. The sequencing data are deposited at ArrayExpress (accession number E-MTAB-6321).

### Downstream bioinformatics analysis

IPA software (content version 24718999, license obtained from Ingenuity Systems, Redwood City, CA) was used to perform canonical pathway analysis, network analysis and upstream regulator analysis^62^. Analysis was performed on all the DEGs (1650 ID mapped genes). Heatmaps for pathways of interest were generated using data output from IPA with genes ordered according to average fold change. For the upstream regulator analysis we focused only on the significant entities (transcription factors and growth factors) that had an overall z-score >2.

### DNA methylation and data analysis

DNA methylation analysis was performed using the Infinium Human Methylation450 Bead chip array at the Bioinformatics and Expression Analysis Core Facility (BEA), Karolinska Institutet, Sweden. The array data were preprocessed and then processed using R statistical programme language version 3.1.2 using RnBeads^63^ (http://rnbeads.mpi-inf.mpg.de/). Analysis with RnBeads included the following steps: (1) loading raw intensity data (idat); (2) prefiltering (removal of SNP-enriched probes, greedycut algorithm and removal of unreliable measurements; (3) normalization (methylumi-noob for background correction^64^; and dasen normalization^65^; (4) quality control including detection and exclusion of technical failures during bisulfite conversion, hybridization, extension and staining); (5) postfiltering (removal of non CpG probes, removal of sex chromosomes). Supplementary Figure 4 shows that the bisulfite conversion was successful. The differential DNA methylation results at CpG sites, gene promoter, whole gene, CpG island and tiling region levels was performed using RnBeads processing pipeline with default settings. DNA methylation data are deposited at ArrayExpress (accession number E-MTAB-6331).

### Migration and invasion assays

Cell migration and invasion assays were performed after 6 weeks of exposure to AgNPs. Cells were harvested using trypsin, re-suspended in additive free medium and counted. For the migration assay, 1.5 × 10^4^ cells were seeded on the upper chamber of a Transwell insert (8 µm pore size, polycarbonate, 0.33 cm^3^ insert surface area) in additive free BEGM medium. For the invasion assay, 3 × 10^4^ cells were seeded on the upper chamber of a BioCoat Matrigel invasion chamber (8 µm pore size, PET, 0.33 cm^3^ insert surface area) in additive free BEGM medium. Thereafter, both migration and invasion inserts were placed in 24 well plates and complete BEGM medium was added in the basal chamber. Cells were allowed to migrate/invade for 48 h at 37 °C in a humidified incubator. Thereafter, cells were fixed with 4% formaldehyde for 15 min, washed and stained with 10% Giemsa (Sigma-Aldrich) for a further 15 min. The non-migrating/non-invading cells were removed from the upper side of the insert using cotton swabs. 5 pictures were taken for each insert using a Nikon ECLIPSE TE2000-S microscope (Nikon Corp) in bright-field mode (100x magnification) to cover the whole insert area. Images were scored and the total number of migrating and invading cells was calculated for each insert. Results are presented as the total number of invading and migrating cells. Additionally, the invasion index (percentage of the migrating cells with the ability to invade) was calculated. TGF-β1 (15 ng/mL for 72 h) (Sigma-Aldrich) was used as a positive control.

### Soft agar cell transformation assay

Anchorage independent cell growth was assessed using the soft agar cell transformation assay. After 3 and 6 weeks of exposure, cell were harvested, re-suspended in cell medium and counted. For the 6-well plate format, 3000 cells were mixed with 1.5 mL 0.35% noble agar prepared in cell medium at 37 °C and added on top of a 1.5 mL 0.7% noble agar solidified base layer. Cells were kept in the incubator and were fed 300 µL cell medium every 3–4 days. After 2.5 weeks transformed colonies were stained with 2% Giemsa (Sigma-Aldrich) and counted. Results are expressed as mean number of colonies per treatment. In parallel, the colony forming efficiency assay was performed and the results were used to account for the plating efficiency and clonogenicity when calculating the transformation index.

### Colony forming efficiency assay

After 3 and 6 weeks of exposure, cells were harvested by trypsinization, counted and diluted 3 successive times. 300 cells were re-seeded in 6 well-plates (in duplicate wells for each sample) and left unexposed during the experiment. The cell medium was changed after 3 days. After 6 days, the cells were washed, fixed in 4% formaldehyde (15 min) and stained with 10% Giemsa (Sigma-Aldrich). The total number of colonies was counted and representative pictures were taken using a Nikon ECLIPSE TE2000-S microscope (Nikon).

### E- and N-cadherin expression

Cell surface expression of E-and N-cadherin was evaluated by flow cytometry. TGFβ1 (15 ng/mL 72 h) (Sigma-Aldrich) was used as a positive control. After 3 and 6 weeks of exposure cells were detached using an enzyme-free cell dissociation buffer (Gibco). Next, cells together with the corresponding supernatants (containing unattached cells) were spun down and re-suspended in 70 µL cell staining buffer (Biolegend). An unstained control sample was prepared by pooling small volumes from all the samples. 5 µL of E-cadherin antibody (Alexa Fluor 488 conjugated, Biolegend) and 5 µL N-cadherin antibody (APC conjugated, Biolegend) were added to each sample (except for the unstained sample) and allowed to incubate on ice, in the dark for 30 min. Next, samples were washed x 2 with cell staining buffer and finally re-suspended in 300 µL of the same buffer. Data was acquired on a BD Accuri C6 flow cytometer (Becton Dickinson). E-cadherin was acquired in FL1 while N-cadherin was acquired in FL4. Data was analyzed using the BD Accuri C6 software. Results were expressed as % E-cadherin positive and % N-cadherin positive cells.

### Collagen analysis

After 6 weeks of exposure (5 days from the last re-seeding) collagen secretion and deposition on the well plate was evaluated using the Sircol assay kit (Biocolor). Briefly, the supernatants were collected in low binding microcentrifuge tubes (Lo-Bind, Eppendorf) while the collagen deposited on the well was extracted with 1.5 mL 0.5 M acetic acid and then collected. Both supernatants and acid extracted samples were concentrated 15 times by incubating with the Isolation & Concentration Reagent at 4 °C over-night. The tubes were then spun down (12000 g, 10 min), and the pellet was mixed with 1 mL Sircol Dye Reagent and placed on a mechanical shaker for 30 min. The samples were then spun down, the collagen-dye pellet was washed with 750 µL Acid-Salt Washed, spun down again, and finally solubilized in 250 µL Alkali Reagent. 200 µL was then transferred in a 96-well plate and absorbance was read at 560 nm using a plate reader (Victor^3^V, Perkin Elmer). A standard curve (0, 1, 2.5, 5, 10, 15 µg collagen) was prepared from collagen reference standard provided in the kit. Soluble collagen secreted in the cell medium was expressed as µg/mL, acid extracted collagen (insoluble collagen deposited on the well plate) was expressed as µg/mL and the total collagen was expressed in µg.

### Genotoxicity assays

Genotoxicity was evaluated by using the comet and micronucleus assays. *Alkaline comet assay*. DNA damage was investigated after 3 and 6 weeks of exposure using the mini-gel version of the alkaline comet assay as previously reported^33^. At least 50 nucleoids were scored per gel with duplicate gels for each sample; triplicate samples were prepared for each exposure. Results are expressed as % DNA in tail and presented as mean ± S.D. (n = 3). *Micronucleus, cell cycle and cytotoxicity by flow cytometry*. The induction of micronuclei and hypodiploid nuclei together with cell cycle alterations and cytotoxicity was determined after 3 and 6 weeks by flow cytometry using the *In Vitro* Microflow Kit (Litron Laboratories) as previously reported^33^. Mitomycin C (0.05 µg/mL, 24 and 48 h) and demecolcin (0.01 µg/mL, 48 h) (Sigma-Aldrich) were used as positive controls for micronucleus induction and cell cycle modulation.

### Statistical analysis

Differences between groups were evaluated by one-way ANOVA followed by Dunnet’s *post hoc* test (for comparisons *versus* control) or by t-test (for comparisons between two groups). For the cell cycle analysis differences between groups were evaluated by two-way ANOVA followed by Bonferroni *post-hoc* test. All analyses were performed using GraphPad Prism version 5.02. p < 0.05 was considered statistically significant.

## Electronic supplementary material

Supplementary information
