# Supplementary material for: RNA-sequencing reveals long-term effects of silver nanoparticles on human lung cells
Source: Sci Rep. 2018 Apr 27;8:6668. doi: 10.1038/s41598-018-25085-5 (PMC5923294; doi:10.1038/s41598-018-25085-5)
Supplement: Supplementary file 1 — Supplementary information [file 41598_2018_25085_MOESM1_ESM.pdf]

SUPPORTING INFORMATION

RNA-sequencing reveals long-term effects of silver nanoparticles on human lung cells

Anda R. Gliga[1], Sebastiano Di Bucchianico[1], Jessica Lindvall[2], Bengt Fadeel[3], and Hanna L. Karlsson[1]

[1]Unit of Biochemical Toxicology, Institute of Environmental Medicine, Karolinska Institutet, Stockholm, Sweden;

[2]National Bioinformatics Infrastructure Sweden, Science for Life Laboratory, Department of Biochemistry and Biophysics, Stockholm University, Stockholm, Sweden;

[3]Division of Molecular Toxicology, Institute of Environmental Medicine, Karolinska Institutet, Stockholm, Sweden.

Running title: Silver nanoparticles induce epithelial-to-mesenchymal transition.

Supplementary Table 1. Significantly enriched pathways Ag10 *vs* Control.

Ingenuity Canonical Pathways	-log(p-value)	z-score
Adipogenesis pathway	4,27	NaN
Atherosclerosis Signaling	3,98	NaN
Axonal Guidance Signaling	3,92	NaN
Gα12/13 Signaling	3,90	0,218
Eicosanoid Signaling	3,15	1,89
STAT3 Pathway	3,08	1,069
Hepatic Fibrosis / Hepatic Stellate Cell Activation	3,00	NaN
Xenobiotic Metabolism Signaling	2,75	NaN
Type I Diabetes Mellitus Signaling	2,53	NaN
LPS/IL-1 Mediated Inhibition of RXR Function	2,48	-0,333
Acute Phase Response Signaling	2,47	-0,728
Aryl Hydrocarbon Receptor Signaling	2,47	NaN
Serine Biosynthesis	2,44	NaN
Glutamate Degradation III (via 4-aminobutyrate)	2,44	NaN
Ephrin Receptor Signaling	2,32	NaN
Leukocyte Extravasation Signaling	2,21	1,091
Wnt/Ca+ pathway	2,13	1,897
SAPK/JNK Signaling	2,04	0
Regulation of the Epithelial-Mesenchymal Transition Pathway	2,03	NaN
Inhibition of Angiogenesis by TSP1	1,96	0,447
Superpathway of Serine and Glycine Biosynthesis I	1,95	NaN
Role of Macrophages, Fibroblasts and Endothelial Cells in Rheumatoid Arthritis	1,91	NaN
p38 MAPK Signaling	1,91	-0,535
p53 Signaling	1,89	0,302
1,25-dihydroxyvitamin D3 Biosynthesis	1,81	NaN
4-aminobutyrate Degradation I	1,81	NaN
Colorectal Cancer Metastasis Signaling	1,79	1,8
Phenylalanine Degradation IV (Mammalian, via Side Chain)	1,78	NaN
Antiproliferative Role of Somatostatin Receptor 2	1,78	1,414
Intrinsic Prothrombin Activation Pathway	1,75	NaN
Human Embryonic Stem Cell Pluripotency	1,69	NaN
PPAR Signaling	1,68	0
Dendritic Cell Maturation	1,66	0
FGF Signaling	1,65	-1,732
Inhibition of Matrix Metalloproteases	1,64	NaN
Glioma Invasiveness Signaling	1,63	-0,333
Role of NFAT in Cardiac Hypertrophy	1,62	0,218
Wnt/β-catenin Signaling	1,60	2,324
Phospholipases	1,59	NaN
PTEN Signaling	1,56	-1,604
Neuregulin Signaling	1,54	1,414
P2Y Purigenic Receptor Signaling Pathway	1,53	1,291
Retinoate Biosynthesis II	1,53	NaN
Phenylethylamine Degradation I	1,53	NaN
Role of MAPK Signaling in the Pathogenesis of Influenza	1,52	NaN
NF-kB Signaling	1,50	-1,342
IL-8 Signaling	1,50	0,655
Gαi Signaling	1,50	2,333
Ovarian Cancer Signaling	1,49	NaN
Clathrin-mediated Endocytosis Signaling	1,48	NaN
HIF1α Signaling	1,42	NaN
Role of IL-17F in Allergic Inflammatory Airway Diseases	1,38	0
Ephrin B Signaling	1,38	0
D-myo-inositol-5-phosphate Metabolism	1,38	NaN
3-phosphoinositide Degradation	1,35	NaN
Thrombin Signaling	1,35	-0,243
NRF2-mediated Oxidative Stress Response	1,35	0,816
Creatine-phosphate Biosynthesis	1,33	NaN
FXR/RXR Activation	1,31	NaN
TR/RXR Activation	1,31	NaN

Pathway analysis was performed in IPA on the differentially expressed genes of the contrast Ag10 *versus* Control. Significantly enriched canonical pathways (-log(p-value>1.3)) are illustrated, ordered according to the statistical significance (-log(p-value)). Some pathways are additionally characterized by z-score, a measure of the activation state of the pathway. NaN, activity pattern not available.

Supplementary Table 2. Upstream regulator analysis by IPA.

Upstream Regulator	Expr Log Ratio	Predicted Activation State	Activation z-score	p-value of overlap
TP53		Activated	2,571	6,61E-20
SMARCA4		Activated	3,035	3,63E-14
CTNNB1			1,05	7,30E-11
GLI1			0,461	7,64E-11
NEUROG1			0,655	9,40E-10
FOS			-0,142	1,91E-09
ERG			1,279	3,09E-09
HIF1A	-0,365	Activated	2,609	1,08E-08
NKX2-3		Activated	2,082	1,59E-08
PAX3			-1,165	4,56E-08
SP1		Activated	2,237	3,82E-07
TAF4			-0,042	5,06E-07
CEBPA			-1,281	7,31E-07
CBX5			-0,816	8,49E-07
CREB1	-0,386		0,107	1,46E-06
TWIST1		Activated	4,084	2,42E-06
STAT3			1,713	2,55E-06
TP73			0,69	3,38E-06
ZEB1		Activated	2,27	3,63E-06
SOX4	-0,705		1,554	4,73E-06
PAX7			-0,842	5,10E-06
ATF4		Activated	3,119	8,62E-06
NFYB	-0,432			1,23E-05
EP300			1,04	1,34E-05
STAT5A			-1,547	1,46E-05
SOX11			0,346	2,18E-05
SP3			1,213	3,20E-05
NFKBIA		Activated	2,089	4,57E-05
MYOCD			-0,344	6,16E-05
KLF2	1,374		1,729	7,35E-05
ZNF217	-0,379		-1,414	8,61E-05
HNF1B			-1,394	9,47E-05
VHL			0,71	9,51E-05
SMAD4			0,117	1,06E-04
PDX1			0	1,20E-04
NCOA4			1,706	1,20E-04
CCND1			-1,897	1,56E-04
BRCA1			0,774	1,65E-04
SIAH2			-1,96	2,37E-04
EHF	-0,851		-0,775	2,56E-04
MYC			-0,529	2,96E-04
MITF			-0,348	3,23E-04
SOX2			1,43	3,33E-04
PRDM1	1,276		-0,632	4,30E-04
TBX5			-0,853	4,47E-04
E2F1		Inhibited	-3,15	5,98E-04
TRPS1			-1,134	6,29E-04
CREBBP			-1,099	6,97E-04
RUNX2			-0,608	7,26E-04
ARNT2			0,962	8,38E-04
TFAP2A			0,794	8,47E-04
HOXC8			-0,707	8,80E-04
PLAG1			0,389	1,06E-03
MYCN			-0,543	1,12E-03
SQSTM1			1,418	1,24E-03
TP63	-2,917		-1,974	1,30E-03
HAND2			-1,129	1,50E-03
CEBPB			-0,573	1,51E-03
NKX2-1				1,62E-03
GTF2I				1,74E-03
NFIB				1,79E-03
MEF2C	0,991		-1,453	2,19E-03
NOTCH1			-0,986	2,22E-03
SNAI1		Activated	2,757	2,54E-03
NFE2L2		Activated	2,023	2,64E-03
CREB3L4				2,85E-03
SRF			0,654	2,92E-03
GATA4			-0,826	3,42E-03
HDAC6			1,555	3,57E-03
EPAS1			0,415	4,40E-03
Sry				4,65E-03
KDM5B			0,256	4,78E-03
NUPR1	1,276		1,286	5,25E-03
SIM1	0,93		0,6	5,33E-03
IKZF1		Inhibited	-2,048	5,77E-03

Upstream Regulator	Expr Log Ratio	Predicted Activation State	Activation z-score	p-value of overlap
MYB			0,745	5,94E-03
CREB3L1	1,879		0,555	5,94E-03
SPDEF			-1,667	6,02E-03
TRIM28			1	6,15E-03
STAT6			0,188	6,27E-03
RELA			-0,262	6,28E-03
SMAD3			0,259	6,37E-03
HTATIP2	0,462			7,61E-03
SNAI2			1,194	7,82E-03
KLF6			0,041	7,85E-03
STAT4			0,949	8,00E-03
SIRT1	-0,39		0,742	8,31E-03
NOTCH3	1,314		1,534	9,00E-03
EGR2		Activated	3,018	9,28E-03
BHLHA15				9,37E-03
MTPN		Activated	2,354	9,46E-03
SMAD7			-1,523	9,49E-03
HDAC2			0,632	9,49E-03
EZH2	-0,368		0,307	9,49E-03
HOXD3			1,4	9,59E-03
ARNT			1,463	1,15E-02
SIN3B				1,19E-02
EGR1			0,962	1,21E-02
POU5F1			-0,102	1,22E-02
PSMC5				1,33E-02
GLI2			-0,676	1,33E-02
LCOR				1,33E-02
PRDM16				1,37E-02
H2AFX				1,45E-02
SKI			-1,387	1,45E-02
PITX2			-0,87	1,50E-02
GRHL2	-2,36		-1	1,60E-02
CNOT7				1,68E-02
ZFP36			1,069	1,70E-02
FOXM1			2	1,70E-02
CDX2			0,208	1,70E-02
KLF4			-0,226	1,90E-02
HMGA1			0,075	2,02E-02
SIX5			0,447	2,10E-02
MECP2			0,426	2,13E-02
HLX			-0,896	2,25E-02
PTTG1			1,455	2,25E-02
ZNF24				2,54E-02
NACA				2,54E-02
KLF5			-0,595	2,63E-02
WWC1	-0,744			2,64E-02
RUVBL2				2,64E-02
Cux1				2,64E-02
HOXA9			0,102	2,72E-02
HTT	0,466		1,814	2,79E-02
LHX2			0	2,90E-02
ZEB2	1,051		1,974	2,90E-02
YBX1			0,197	2,91E-02
GLIS2		Inhibited	-2	3,05E-02
MAX			1	3,17E-02
YAP1				3,17E-02
SATB1			-0,276	3,28E-02
MEN1			-0,817	3,33E-02
POU2F1				3,41E-02
LMX1B				3,46E-02
FOXD3				3,46E-02
ID4				3,46E-02
EBF2			1,192	3,67E-02
CALR			0,555	3,76E-02
TCF7L2			-0,895	3,76E-02
MSC	1,698		1,414	3,86E-02
MKX				4,05E-02
MAFK				4,34E-02
SATB2				4,38E-02
PRDM8	1,428			4,38E-02
MEF2A			0	4,43E-02
HOXA7			-1,342	4,43E-02
RB1			0,71	4,86E-02
SREBF1			-0,372	4,87E-02
GFI1			1,827	5,16E-02

Upstream regulator analysis was performed in IPA for transcription factors. Some of these molecules were also differentially expressed (indicated by the expr log ratio column). The activation state is predicted from the z-score.

Supplementary Table 3. Significantly enriched gene ontologies.

	Level	q	p
**BIOLOGICAL PROCESS**			
angiogenesis	10	49	1,85E-12
cell adhesion	3	110	4,28E-11
regulation of endothelial cell proliferation	6	20	8,08E-10
extracellular matrix organization	4	46	1,96E-09
positive regulation of signaling	4	111	8,77E-09
blood coagulation	5	55	1,31E-08
chemotaxis	4	69	6,80E-08
negative regulation of transmembrane receptor protein serine/threonine kinase signaling pathway	7	19	3,13E-07
negative regulation of epithelial cell proliferation	6	18	5,36E-07
glycosaminoglycan biosynthetic process	6	19	5,98E-07
negative regulation of cellular response to growth factor stimulus	5	19	7,21E-07
cell growth	3	13	9,12E-07
dicarboxylic acid catabolic process	8	7	1,30E-06
positive regulation of epithelial cell proliferation	6	22	1,97E-06
collagen metabolic process	5	16	3,23E-06
glycosaminoglycan catabolic process	6	13	3,42E-06
regulation of peptidase activity	6	34	4,12E-06
cell migration	5	81	4,59E-06
canonical Wnt receptor signaling pathway	7	10	4,76E-06
positive regulation of ion transport	6	22	5,44E-06
**CELLULAR COMPONENT**			
cytosol	7	253	8,67E-27
integral to plasma membrane	7	142	8,61E-19
extracellular region part	2	147	1,87E-17
extracellular matrix	1	80	2,01E-14
endomembrane system	3	194	2,81E-13
cell surface	3	73	2,83E-12
cell projection	3	165	3,92E-12
perinuclear region of cytoplasm	7	64	1,22E-09
plasma membrane	4	444	2,78E-09
lysosome	9	48	3,64E-08
nuclear membrane	10	33	2,13E-07
vacuolar lumen	9	13	1,96E-05
cytoplasmic membrane-bounded vesicle	8	99	3,50E-05
apical part of cell	3	41	3,67E-05
leading edge membrane	6	16	4,60E-05
cell junction	1	87	4,77E-05
cytoplasm	5	891	0,00016
platelet alpha granule	10	10	0,00025
membrane raft	3	27	0,00039
Z disc	11	16	0,00041
**MOLECULAR FUNCTION**			
protein binding	2	725	9,60E-66
glycosaminoglycan binding	3	34	8,21E-11
metal ion binding	4	381	6,30E-10
sulfur compound binding	2	31	2,00E-09
transcription cofactor activity	3	51	6,17E-06
protein phosphatase binding	5	14	2,11E-05
lipid binding	2	99	2,66E-05
aldo-keto reductase (NADP) activity	5	6	6,24E-05
monocarboxylic acid binding	5	11	6,36E-05
tumor necrosis factor-activated receptor activity	6	5	0,00015
receptor signaling protein activity	3	17	0,00032
steroid hormone receptor binding	6	10	0,00034
ubiquitin protein ligase binding	5	17	0,00092
extracellular matrix structural constituent	2	12	0,00098
RNA polymerase II core promoter proximal region sequence-specific DNA binding transcription factor activity involved in positive regulation of transcription	5	12	0,00143
structural constituent of muscle	2	7	0,00205
GDP binding	8	7	0,00230
mRNA binding	5	14	0,00234
acetylgalactosaminyltransferase activity	5	6	0,00252
Wnt-activated receptor activity	5	5	0,00280

Gene ontology enrichment was performed as described in Methods. Top20 ontologies for each domain are illustrated, ordered according to the p-value. The cutoff for p-value was set at 0.05. (q, number of gene in ontology; p, p-value of enrichment)

Supplementary Table 4. DNA methylation analysis (Illumina 450k)

Ag *vs* Control	# FDR adjusted p-value < 0.05	Site/Gene/Region ID	Chromosome	Start	End	Mean Difference Methylation Ag vs Control	Location of the region/site of interest
**methylated site**	6	cg21242448	chr9	120510294		-0.88	intergenic region, S-shelf of CpG island
		cg05712748	chr1	43472312		0.43	intergenic region, N-shore of CpG island
		cg25673075	chr5	111963982		-0.74	intergenic region, proximity of novel lincRNA ENSG00000250358
		cg23492225	chr10	2147217		0.33	intergenic region
		cg17858192	chr4	16077807		-0.42	5'UTR region, ENSG00000007062 (PROM1)
		cg04993605	chr1	28573052		-0.34	H3K27Ac Mark (often found near active regulatory elements)<br>Binding site for transcription factors EP300, POLR2A, TEAD4, GATA3, SMC3, CEBPB, FOXA1, STAT3, FOXA2, FOS, MYBL2, ELK4, SIX5, NFATC1, FOXM1, NFIC
**promoter level**	1	ENSG00000250358	chr5	111962633	111964632	-0.74	novel lincRNA
**tiling regions**	5	331939	chr9	120510001	120515000	-0.88	intergenic region
		198720	chr5	111960001	111965000	-0.74	intergenic region, proximity of novel lincRNA ENSG00000250358
		220774	chr6	41310001	41315000	-0.45	protein coding gene ENSG00000096264<br>region contains biding sites for transcriptions factors EBF1, TCF3, SP1, TCF12
		377076	chr11	69445001	69450000	0.30	intergenic region<br>region contains binding sites for SETDB1, CBX3, SIX5, NFYB, MAX, USF2, USF1, ATF3, ZNF143, CEBPB
		251232	chr7	22480001	22485000	0.52	H3K27Ac Mark (often found near active regulatory elements)<br>region contains binding sites for EZH2, SIN3AK20, ARID3A, MXI1, REST, FOXA1, TEAD4, HNF4G, FOXA2, NFIC, EP300, HDAC2, MYBL2, RXRA, HNF4A, ZNF217, SP1, EGR1, ZNF263, GATA3, GATA2, FOXA1, MYC, MBD4, POLR2A, CCNT2, SIRT6, RCOR1, FOS, JUND, CBX3, ATF1, HNF4G, TBP, ELF1, MAX, E2F6, BHLHE40, FOSL2, YY1, USF1, SPI1, POLR2A, ESR1, IRF4, RELA, JUN, BCL11A, NR3C1, EBF1, RUNX3, MTA3, IKZF1, CTCF, FOXM1, EBF1, PBX3, TAF1, TCF12, TCF3, MEF2A,
**gene**	0						
**CpG Island**	0						

DNA methylation analysis following 6 weeks of BEAS-2B exposure to 1 µg/mL to Ag10 was performed using Illumina 450k. Differential methylation analysis was performed using the Bioconductor package RBeads for methylated sites, promoter level, tiling regions (regions with a window size of 5 kilobases), genes and CpG islands. Location data are derived from the UCSC and Ensembl genome browsers.

A

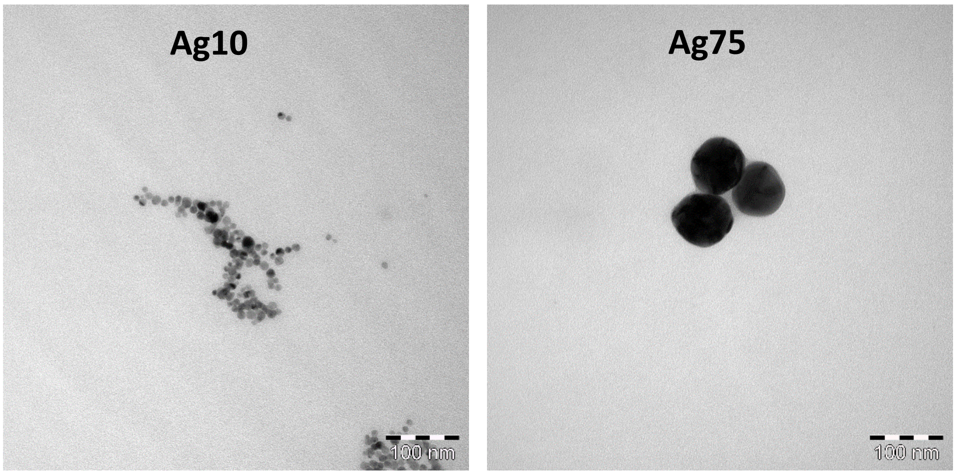

B

	Time point (h)	Approx. size peak max (nm)	Scattered light intensity (average, kcps)	Zeta-potential (average, mV)
**Ag10**	0 h	10, 1000	730	-0.587
	24 h	10, 100, 1000	63	N.D.
**Ag75**	0 h	3.5, 40, 400	2790	-7.78
	24 h	5, 100, 1000	1100	N.D.

C

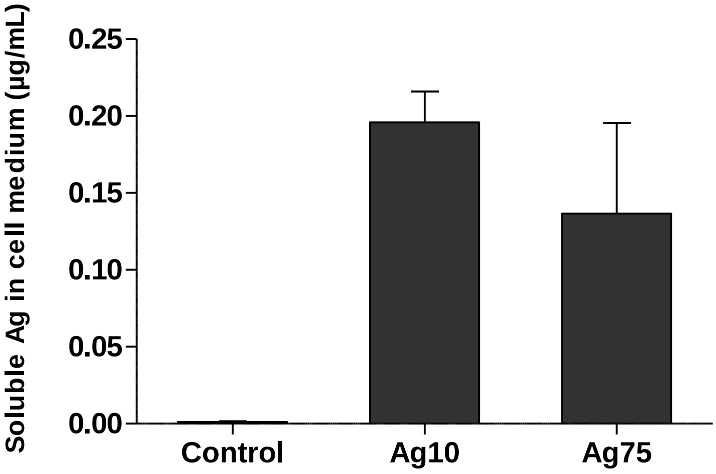

Suppl. Figure 1. **Characterization of AgNPs. (A)** Primary particle size was confirmed using TEM. **(B)** Hydrodynamic size-distribution in BEGM (data from Gliga *et al* 2014) and zeta potential values in BEGM medium. **(C)** Soluble Ag content in cell medium investigated by ICP-MS. Cell supernatants were harvested 4 days after exposure to 1 µg/mL AgNPs and centrifuged 15000 rpm for 30 min. 150 µL supernatant was carefully collected and analyzed by ICP-MS as described in Methods. Results are presented as mean soluble silver (µg/mL) ± S.D. (n=3).

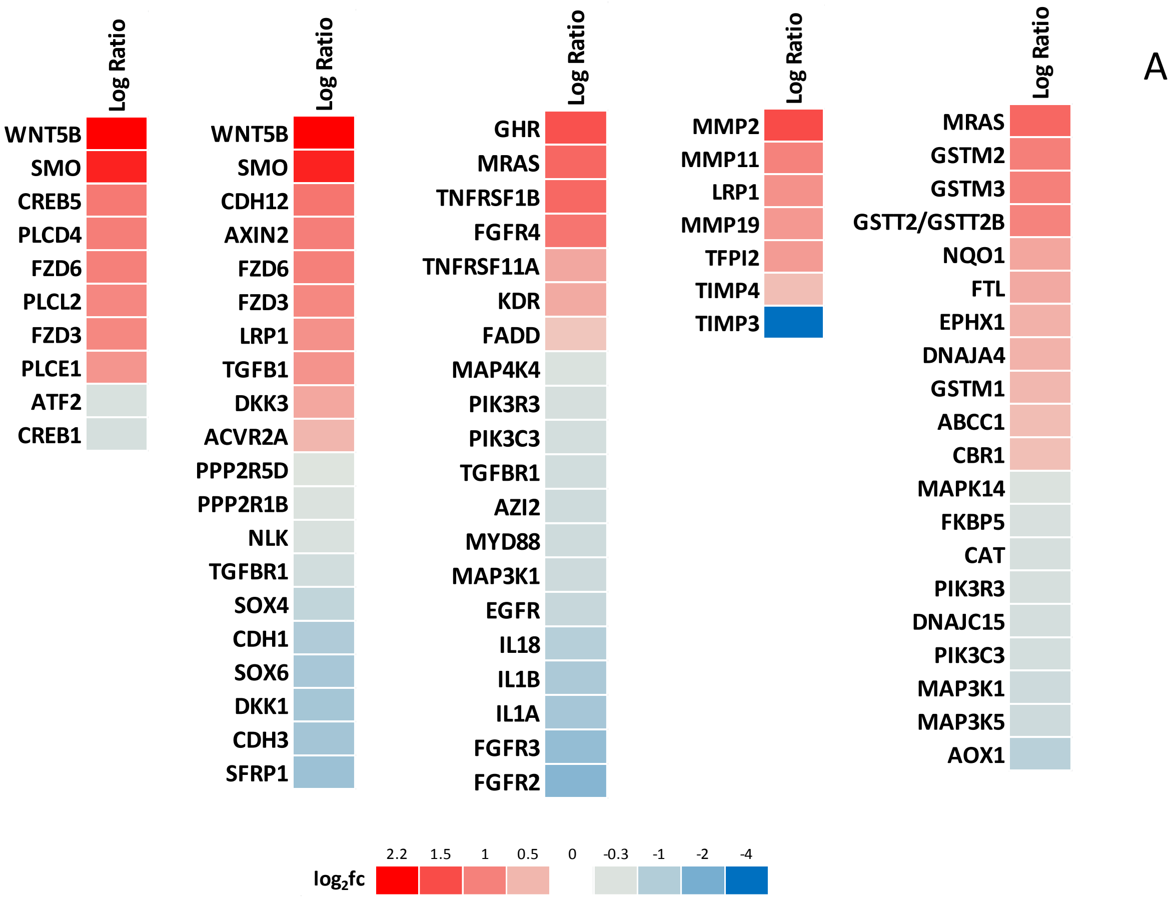

Suppl. Figure 2. Ingenuity Pathway Analysis identified enriched pathways after 6-week exposure of BEAS-2B cells to Ag10. Using the IPA tool, we performed downstream pathway analysis of the DEGs for Ag10. Heatmaps displaying the gene expression changes in the Ag10 vs Control were generated for the Wnt/Ca^{2+} pathway (A), Wnt/βcatenin signalling (B), NF-κB signalling (C), Inhibition of Matrix Metalloproteases (D) and NRF-2 mediated Oxidative Stress response (E).

A

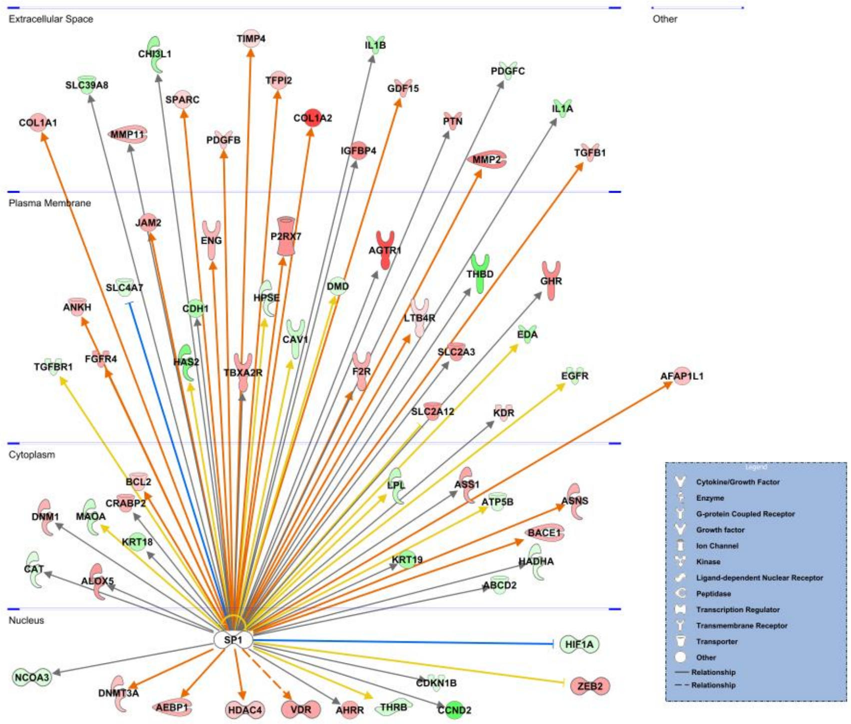

B

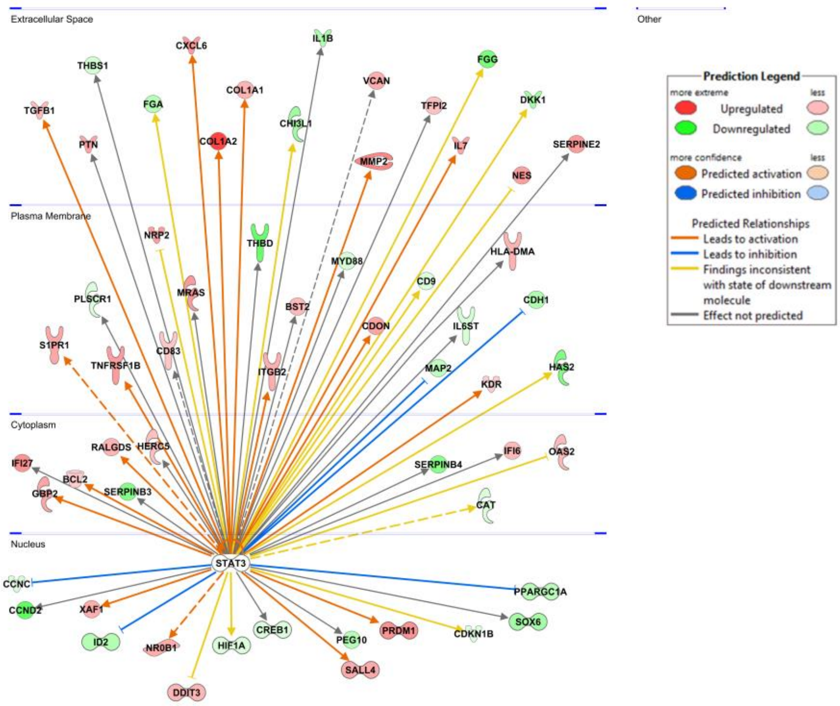

Suppl. Figure 3. SP1 and STAT3 transcription factors. Upstream regulator analysis was performed using IPA for Ag10 and a network was generated around the predicted top 2 transcription factors SP1 (A) and STAT3 (B) which had an absolute z-score higher than 1.5 and were also found to bind differentially methylated sites or regions in the DNA methylation assay.

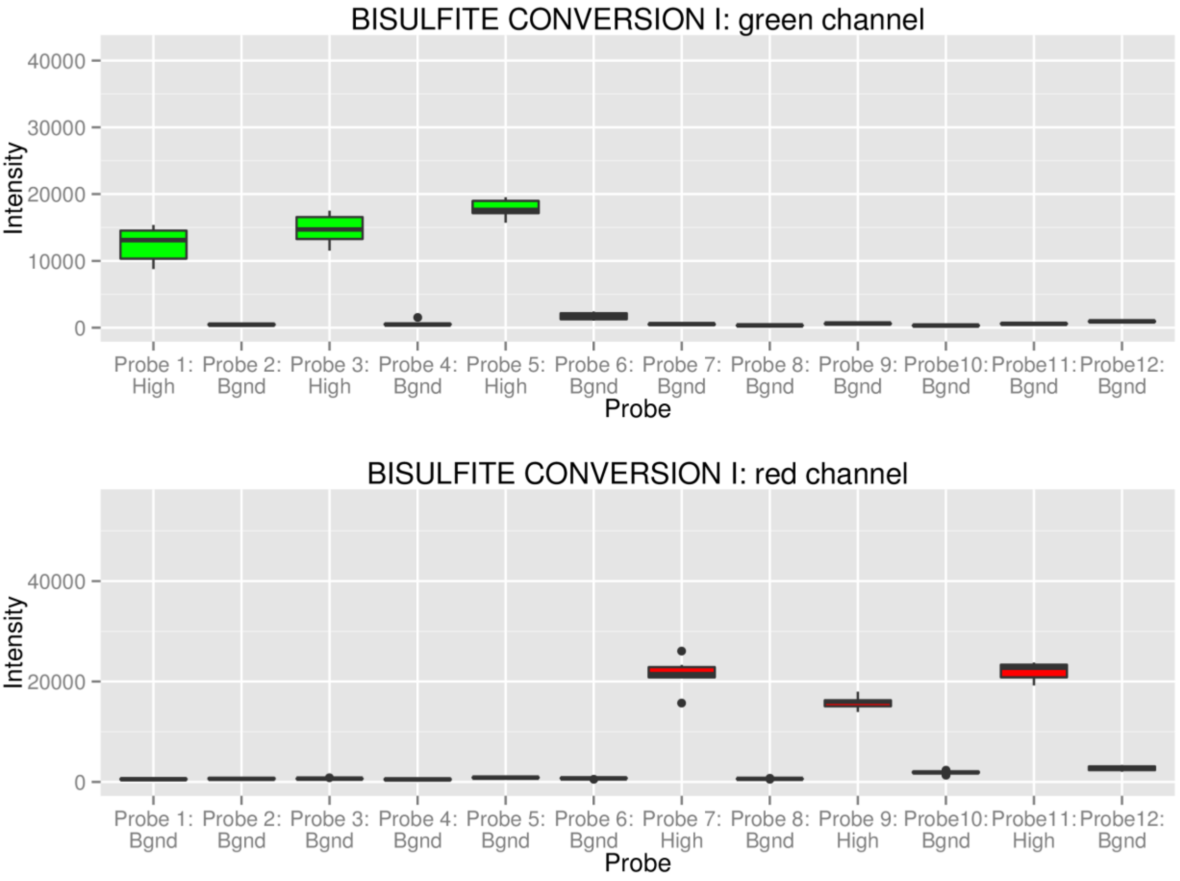

Suppl. Figure 4. Box-plot showing bisulfite conversion to test the efficiency of bisulfite conversion by query of C/T polymorphism. The green output shows Type I bisulfite conversion and the red channels reflects the reading of the type II bisulfite conversion data. Overall output showed that the bisulfite conversion was successful and, hence, the DNA methylation worked as expected.

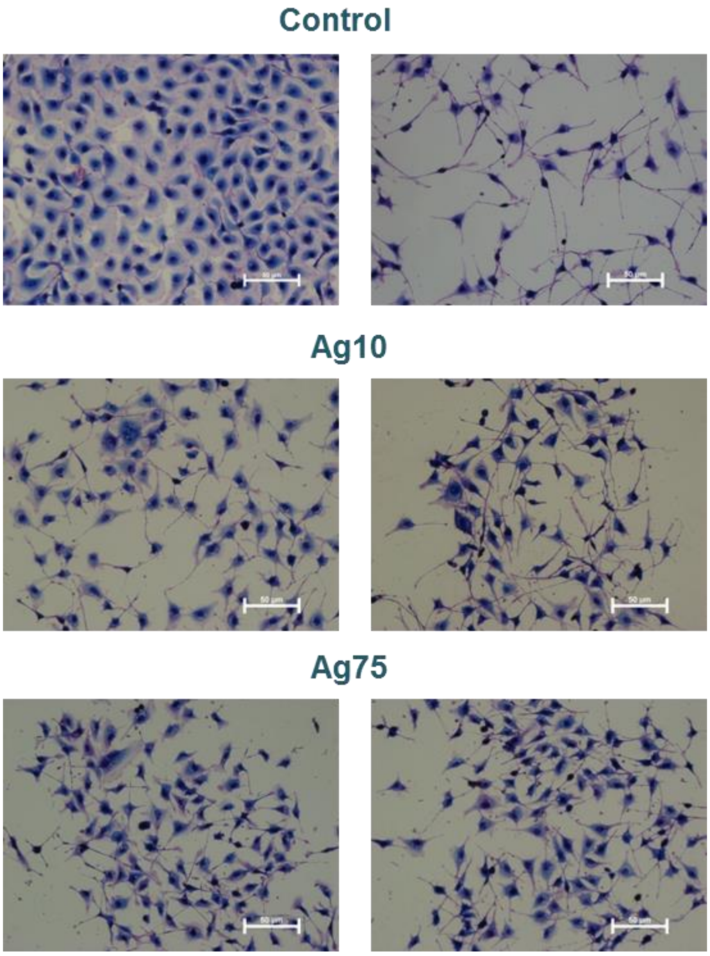

Suppl. Figure 5. Colony forming efficiency. Representative images of the colonies are included. Two distinct colony phenotypes were observed for the control (dense colonies with high cell-cell contact and sparse colonies with a low cell-cell contact) whereas the Ag treated cells displayed a more homogenous colony phenotype (mostly sparse colonies with a low cell-cell contact).
